# Role of PARP-1 in Human Cytomegalovirus Infection and Functional Partners Encoded by This Virus

**DOI:** 10.3390/v14092049

**Published:** 2022-09-15

**Authors:** Wenchang Zhang, Jing Guo, Qiang Chen

**Affiliations:** Frontier Science Center for Immunology and Metabolism, Medical Research Institute, Wuhan University, Wuhan 430071, China

**Keywords:** HCMV, PARP-1, PARG, PARylation, UL76, RGG motif

## Abstract

Human cytomegalovirus (HCMV) is a ubiquitous pathogen that threats the majority of the world’s population. Poly (ADP-ribose) polymerase 1 (PARP-1) and protein poly (ADP-ribosyl)ation (PARylation) regulates manifold cellular functions. The role of PARP-1 and protein PARylation in HCMV infection is still unknown. In the present study, we found that the pharmacological and genetic inhibition of PARP-1 attenuated HCMV replication, and PARG inhibition favors HCMV replication. PARP-1 and its enzymatic activity were required for efficient HCMV replication. HCMV infection triggered the activation of PARP-1 and induced the translocation of PARP-1 from nucleus to cytoplasm. PARG was upregulated in HCMV-infected cells and this upregulation was independent of viral DNA replication. Moreover, we found that HCMV UL76, a true late protein of HCMV, inhibited the overactivation of PARP-1 through direct binding to the BRCT domain of PARP-1. In addition, UL76 also physically interacted with poly (ADP-ribose) (PAR) polymers through the RG/RGG motifs of UL76 which mediates its recruitment to DNA damage sites. Finally, PARP-1 inhibition or depletion potentiated HCMV-triggered induction of type I interferons. Our results uncovered the critical role of PARP-1 and PARP-1-mediated protein PARylation in HCMV replication.

## 1. Introduction

Herpesviruses shared a unique four-layered structure including linear double-stranded DNA genome packaged in an icosahedral capsid which is surrounded by an amorphous protein layer known as the tegument. The tegument is further enclosed in a glycoprotein-containing lipid bilayer [1]. The genome of herpesviruses is temporally expressed in the permissively infected cells with a cascade of immediate early (IE), early (E) and late (L) genes. Based on the cell tropism, growth kinetics and cytopathic effect, the herpesviruses are further divided into three subfamilies, the α-, β- and γ-herpesviruses. The β-herpesvirus exhibit relatively lengthy replication cycles, remain substantially cell-associated, and generally do not cross host–species barriers [2]. Human cytomegalovirus (HCMV), the prototypical β-herpesvirus, is a major cause of morbidity and mortality in immunocompromised individuals. HCMV is also one of the leading infectious agents causing congenital infection [3].

Timescales needed for viruses to finish their replication cycles differ from each other. For instance, human simplex virus 1 (HSV-1) finished a replication cycle in fibroblasts or epithelial cells within about 24 h, whereas HCMV did not achieve maximal yields until about 96 h post-infection [4]. That means avoiding premature cell death was extremely important for HCMV-productive infection. HCMV infection potently induced cytokines’ secretion [5], TLRs’ activation [6], DNA damage responses [7,8] and ER stress [9]. All of these stimuli triggered programmed cell death, whereas the protracted replication cycle forces HCMV to maintain host-cell viability for a relatively long period of time. To achieve this, HCMV has evolved mechanisms to manipulate host processes, especially to interfere with the induction and execution of programmed cell death, for production of progeny virus [10,11].

UL76 is a Herpes_UL24 family (PF01646) member in HCMV that has been shown to display multiple functions. Members of the Herpes_UL24 family share some common features, such as they all have a putative PD-(D/E)XK endonuclease motif [12]. Based on multiple protein-sequence alignments, members of this family were found to contain five conserved amino acid blocks at the N terminus (1–190 aa of UL76) and a variable sequence at the C terminus (191–325 aa of UL76). The HCMV UL76 protein is virion-associated and expressed with true late kinetics [13]. The most significant feature of UL76 is that it contains many positive-charged residues with a putative pI of 11.6. UL76 presents as globular aggresomes in the nuclei of transiently transfected cells [14]. In cells presenting long-term expression of UL76, the hallmark of chromosome aberrations and abnormal mitosis was observed [15]. The interaction between UL76 and S5a, which is a major receptor of polyubiquitinated proteins for UPS proteolysis, was associated with UL76′s ability to induce nuclear aggresomes’ formation [14]. Global mutational analysis defined UL76 as an essential or augment gene for virus reproduction [16,17]. However, in the UL76 stably expressed human glioblastoma, HCMV production was significantly delayed [13]. Thus, the function of UL76 on HCMV infection deserved further investigation.

The human genome encodes as many as 17 members of the poly (ADP-ribose) polymerase (PARP) family, characterized by their ability to transfer negatively charged ADP-ribose groups from donor nicotinamide adenine dinucleotide (NAD^+^) molecules onto target proteins post-translationally (PARylation). Among which, PARP-1 was the founding member of this family and accounts for more than 90% of PARP activity in the cell nucleus [18]. Protein PARylation has been implicated in many important cellular processes, such as DNA repair and replication, mitosis, modulation of chromatin structure and other stress signaling [19,20,21,22,23]. PARP-1 overactivation obviously decreases the cellular NAD^+^ level and causes energy depletion through PAR-dependent inhibition of glycolysis [24]. The cellular level of poly (ADP-ribose) (hereafter PAR) was regulated by PAR polymerases (PARPs) and the degrading enzyme poly (ADP-ribose) glycohydrolase (PARG), controlling the cell-fate decision between life and death in response to genetic insults. PAR accumulation is transient after DNA damage. PAR is rapidly degraded by PARG, an enzyme with both exo- and endo-glycosidase activities that generates large amounts of free ADP-ribose [25]. Human PARG is encoded by a single gene. Alternative splices give rise to PARG isoforms target to different cell compartments [26]. The production and accumulation of PAR, which reflects the severity of the DNA damage, directly contributes to the cellular decision to initiate either survival- or death-programs.

PARP-1 has been implicated in the infection and pathogenesis of several viruses [27,28,29]. Both antiviral and proviral roles of PARP-1 in viral infection have been reported, which depends on the types of viruses. For example, PARP-1 is necessary for efficient integration of the human immunodeficiency virus 1 (HIV-1) proviral genome [30]. The interaction between HBx of hepatitis B virus (HBV) and PARP-1 was implicated in HBV-associated hepatocarcinogenesis [31]. E4orf4 encoded by adenovirus interacts with PARP-1 and inhibits protein PARylation. PARP-1 inhibition enhanced adenovirus replication [32]. The PARP-1 activity on HSV-1 replication depends on the concentration and what kinds of inhibitors are tested; a low concentration of Olaparib treatment has no effect on HSV-1 replication, while a high concentration of 3-aminobenzamide (3-ABA, a broad-spectrum ADP-ribosylation inhibitor) caused a moderate decrease in viral yield [33]. PARP-1 regulated the latency of the Epstein–Barr virus (EBV) and Kaposi sarcoma-associated herpesvirus (KSHV) by PARylating Epstein–Barr nuclear antigen 1 (EBNA1) and latency-associated nuclear antigen (LANA) and inhibiting the binding of these proteins to viral replication origin, respectively [34,35]. In addition to disrupting the latency of KSHV, PARP-1 interferes with the reactivation of this virus. PARP-1 specifically interacted with the replication and transcription activator (RTA) of KSHV, decreasing its recruitment to the promoter region and disrupting the reactivation of KSHV [36]. To overcome the repressive effect of PARP-1 on RTA, two conserved γ-herpesvirus genes functioned through different ways. On the one hand, PF-8, a viral processivity factor, recruits CHFR for degradation of PARP-1 through direct binding to PARP-1 [37,38]. On the other hand, the conserved ORF49 of murine gammaherpesvirus 68 (MHV-68) and KSHV binds to RTA and PARP-1, disrupts the interaction between RTA and PARP-1 and enhances the RTA promoter activity [39,40]. However, the role of PARP-1 and PARP-1-mediated protein PARylation on HCMV replication and virus encode PARP-1 partners were less studied. In the present study, we investigated the role of PARP-1 and PARG on HCMV infection and identified HCMV UL76 as a novel regulator of PARP-1 activity.

## 2. Materials and Methods

### 2.1. Cells and Virus

HEK293T (National Collection of Authenticated Cell Cultures, SCSP-502), HeLa (China Center for Type Culture Collection, GDC0009), U2OS (American Type Culture Collection, HTB-96) and RPE (American Type Culture Collection, CRL-4000) cells were cultivated in Dulbecco’s minimal essential medium (DMEM) (Yuanpei, Shanghai, China) containing 10% fetal bovine serum, 100 U/mL penicillin and 100µg/mL streptomycin. Primary human lung fibroblasts MRC-5 (American Type Culture Collection, CCL-171) were maintained in Eagle’s minimal essential medium (MEM) (Yuanpei, Shanghai, China) supplement with 10% fetal bovine serum. RPE cells with inducible expressing SFB-tagged HCMV UL76 were obtained by lentivirus (pCW-SFB-UL76-puro) transduction and puromycin (5 µg/mL) selection. Due to the limited life-span of the MRC-5 cells, human telomerase reverse transcriptase (hTERT)-immortalized Human Foreskin Fibroblasts (HFFs-hTERT) were employed to construct PARP-1 knockout fibroblasts. PARP-1 knockout 293T, HeLa and HFFs-hTERT cells were generated by CRISPR-cas9 technology. Two individual guide RNAs 5′-CACCGTTGATGGAAAAGTCCCACAC-3′ and 5′-CACCGCAGAAAGTCAAGAAGACAG-3′ were designed and cloned into CRISPR-V2 vector. The 293T and HeLa cells were transfected with gRNA expression plasmid. HFFs-hTERT were infected with PARP-1 gRNA expression lentivirus. HeLa and 293T cells were then cultured for 48 h in the presence of 2 μg/mL puromycin. HFFs-hTERT were cultured for 48 h in the presence of 5 μg/mL puromycin which was used as the selection agent. After serial dilution into 96-well plates, the resulting single clones were subjected to Western blot for analysis of PARP-1 knockout efficiency.

HCMV AD169 strain (ATCC-VR538) was propagated in MRC-5 cells. One day before virus inoculation, 3 × 10^6^ cells were seeded into a 10 cm dish. On the second day, the medium was removed and the cells were washed with PBS. MRC-5 cells were infected with HCMV at an MOI of 0.01–0.02 for 1 h at 37 °C, then the cells were refed with fresh medium. On day 4, culture medium was replaced with MEM containing 5% fetal serum and sustained for another 4 days. Cell-free virus stocks were prepared by centrifugation (2000 rpm for 20 min and supernatant was collected). Virus stocks were titrated by standard plaque formation assay with some modifications. In short, the MRC-5 cells were seeded in 24-well plate with a concentration of 5 × 10^4^ cells per well, one day before titrating. Virus stocks were series diluted in MEM and infected two wells each dilution. During the adsorption for 1 h, cover medium was prepared by mixing 21 mL of prewarmed MEM (10% fetal bovine serum) with 4 mL sterilized 2% agarose and 250µL 5 M Tris pH7.4. After adsorption, the cover medium was added to the well. Plaques were calculated between 7–9 days post-infection. UV-inactivated HCMV was achieved by exposure of HCMV to UV light (5 J/cm^2^) in a HL-2000 UV cross-linker (UVP), and sodium pyruvate was added to a final concentration of 5 mM to prevent damage from free radicals induced by UV radiation.

### 2.2. Reagents

PARP-1 inhibitor Olaparib (MCE (Shanghai, China), HY-10162) and PARG inhibitor PDD 00017273 (MCE, HY-108360) were dissolved in dimethyl sulfoxide (DMSO) and used at a final concentration of 10 μM. Phosphonoformic acid (MCE, HY-B1318) was dissolved in water used at a final concentration of 250 μg/mL. Nicotinamide adenine dinucleotide (NAD^+^) (MCE, HY-B0445) was dissolved in water.

### 2.3. Oligonucleotides and Plasmid Construction

The primers used for plasmid construction are listed in Table 1.

### 2.4. NAD^+^ Measurements

NAD^+^ levels were measured using a NAD^+^/NADH quantification KIT (Beyotime (Beijing, China), S0175) with WST-8 according to the manufacturer’s instructions. Briefly, about 1 × 10^6^ MRC-5 or RPE cells were collected and lysed in 200 μL of NAD^+^/NADH extraction buffer for 20 min on ice. Whole cell extracts were clarified by centrifugation for 10 min at 12,000 *g*. The suspension was used to measure total NAD^+^/NADH. Half of the cell extract was transferred to a new tube and incubated at 60 °C for 30 min to decompose the NAD^+^ while NADH was left. Total NAD^+^/NADH or NADH samples were added to a 96-well plate at 20 μL/well. Subsequently, 90 μL of alcohol dehydrogenase solution was added and incubated at 37 °C for 10 min. Finally, 10 μL of chromogenic solution was added to each well and the mixture was incubated at 37 °C for 30 min. The absorbance values were measured at 450 nm. The amount of NAD^+^ was calculated by subtracting NADH from total NAD^+^/NADH. The relative NAD^+^ levels were normalized to the protein content of each sample.

### 2.5. Western Blot

Cells were lysed in NETN 420 buffer (20 mM Tris-HCl, 420 mM NaCl, 0.5% NP40, 1 mM EDTA) on ice for 20 min with intermittent shaking at 5 min intervals. Cell lysates were centrifuged at 15,000 rpm (4 °C) for 15 min. Protein concentrations were measured with a BCA kit following the manufacturer’s instructions. Cell lysates were separated by SDS-PAGE and transferred to polyvinylidene difluoride membranes (Immobilon-P membranes; Millipore). Membranes were blocked with blocking buffer (5% skim milk in TBST) for 1 h at room temperature. Primary antibodies and their dilution used in these experiment were: anti-PARP-1 (Proteintech (Wuhan, China), 13371-1-AP, 1:3000), anti-PARG (Proteintech, 27808-1-AP, 1:3000), anti-PAR (Trevigen (Gaithersburg, MD, USA), 4335-MC-100, 1:1000) and Abcam (Cambridge, UK), ab14459, 1:1000), anti-γH2AX (Abcam, ab81299, 1:1000), anti-H3 (Proteintech, 17168-1-AP, 1:3000), anti-pp65 (Abcam, ab49214, 1:3000), anti-IE1 (Abcam, ab30924,1:3000), anti-HSV-1 gC (Abcam, ab8230, 1:1000), anti-UL44 (Santa Cruz(Dallas, TX, USA) sc-69744,1:1000), anti-actin (ABclonal (Woburn, MA, USA), AC026, 1:3000), anti-FLAG (Sigma-Aldrich (Burlington, MA, USA)), F2555, 1:10,000), anti-myc (Biolegend (San Diego, CA, USA)), 626801, 1:3000), anti-beta tubulin (Proteintech, 10094-1-AP, 1:3000). After being incubated with primary antibodies for 1 h at RT, the membranes were subsequently washed with TBST for 4 times. Membranes were probed with horseradish peroxidase-conjugated secondary antibodies and the proteins were visualized by chemiluminescence reagents. For histone extraction, cells were firstly lysed with NETN 100 buffer (20 mM Tris-HCl, 100 mM NaCl, 0.5% NP40, 1 mM EDTA) for 20 min on ice. The nucleus was pelleted and washed with water before extraction with newly prepared 0.2 N HCl. Extraction was performed on ice for 1 h. Nuclear extracts were neutralized with 1/10 volume of 2 N NaOH.

### 2.6. Cell Fraction

MRC-5 cells in 6 cm culture dishes were infected or mock infected with HCMV at an MOI of 1 for 48 h. The cytosolic and nuclear fractions were obtained according to documented methods [41]. Briefly, cells were lysed in hypotonic buffer (10 mM HEPES, pH 7.9, 2 mM MgCl_2_, 100 μM EDTA, 10 mM KCl, 0.2% NP40, 1 mM DTT) supplement with protease inhibitor on ice for 30 min. The cell lysates were separated by centrifugation for 5 min at 3000 rpm and the supernatants were collected as the cytosolic fractions. The pellets were washed with 1 mL wash buffer (10 mM HEPES (pH 7.9), 2 mM MgCl_2_, 20 mM KCl, 100 μM EDTA) for 5 times. The pellets then were resuspended in 80 μL hypertonic buffer (20 mM HEPES (pH 7.9), 1.5 mM MgCl_2_, 640 mM NaCl, 200 μM EDTA, 2.5% Glycerol) supplemented with protease inhibitor cocktail. Following 3 rounds of high-speed vortex and centrifugation at 13,000 rpm for 15 min, the supernatants were collected as the nuclear fractions. Both the cytoplasmic and nuclear fractions were denatured in 5 × SDS loading sample buffer and boiled for 5 min.

For detection of the chromatin-bounded PARP-1, the cells in 6 cm dishes were firstly lysed in 200 μL NETN 300 (20 mM Tris-HCl pH8.0, 300 mM NaCl, 1 mM EDTA, 0.5% NP40) buffer supplemented with protease inhibitor for 30 min on ice. Following centrifugation at 12,000 rpm for 10 min, the pellets were washed with 1 mL of low stringency buffer (2 mM EDTA, 1 mM EGTA, 10 mM MgCl_2_) for 5 times. The cell pellets were centrifuged at 12,000 rpm for 5 min and resuspended with 60 μL of Benzonase buffer (Benzonase (Millipore, E1014) 500 U/mL, 20 mM Tri-HCl pH 8.0, 420 mM NaCl, 0.5%NP40, 1 mM MgCl_2_) and incubated for 1 h. After centrifugation, the supernatants were collected as chromatin-bounded fractions.

### 2.7. Immunoprecipitation

Human embryonic kidney 293T cells were transiently transfected using Lipofectamine 2000 (Invitrogen, Waltham, MA, USA). Cells were co-transfected with SFB-tagged wild-type or mutant PARP-1 combined with myc-tagged UL76 constructs or GFP-tagged wild-type or mutant PARP-1 combined with SFB-tagged UL76. Following transfection (48 h), the cells were lysed in NETN420 buffer supplemented with 500 U/mL Benzonase and protease inhibitors. Whole cell extracts were clarified by centrifugation and Na^+^ concentration was adjusted to 170 mM with NETN buffer (20 mM Tris-HCl pH 8.0, 0.5% NP40, 1 mM EDTA). The cell lysates were incubated with streptavidin beads for 4 h at 4 °C. Following 3–5 times washing steps with NETN100 buffer, the bounded proteins were boiled in SDS-PAGE loading buffer and analyzed by immunoblotting. For the detection of the interaction between the endogenous PARP-1 and the virus-encoded UL76, MRC-5 cells were infected or mock infected with HCMV at an MOI of 1 for 96 h. Since UL76 expressed with true late kinetics, the mock-infected cells were collected at 48 hpi and the infected MRC-5 cells were collected at 96 hpi. Clarified cell lysates were subjected to immunoprecipitation with IgG or PARP-1 antibody. The presence of UL76 in the immunocomplex was analyzed by Western blot.

### 2.8. Laser Micro-Irradiation and Live Cell Image

U2OS or HeLa cells were seeded on glass-bottomed dishes. One day before irradiation, the cells were transfected with the indicated plasmid using Lipofectamine 2000 (Thermo Scientific, Waltham, MA, USA) according to the manufacturer’s instructions. Laser micro-irradiation was performed by using an IX 71 microscope (Olympus, Tokyo, Japan) coupled with the MicroPoint Laser Illumination and Ablation System (Andor, Belfast, UK). A 337-nm laser diode (3.4 mW) transmits through a specific dye cell and then yields a 365 nm wave length (UVA) laser beam that is focused through a ×60 UPlanSApo/1.35 oil objective. Images were captured at 20 s intervals.

### 2.9. Immunofluorescence

WT or PARP-1 KO HeLa cells grown on glass-bottomed culture dishes were transfected with GFP-UL76. On the second day, the GFP positive cells were irradiated and fixed in 4% paraformaldehyde for 30 min. Cells were permeabilized with 1% Triton X-100 in PBS for 30 min and blocked with 10% FBS in PBS. First, the antibodies were diluted in blocking buffer. Following co-staining with the PAR (mouse, 1:400, Trevigen, 4335-MC-100) and γH2AX (rabbit, 1:200, Abcam, ab81299) antibodies, the cells were incubated with TRITC-conjugated goat anti-mouse IgG (H + L) and Cy5-conjugated goat anti-rabbit IgG (H + L) secondary antibodies. Images were taken by microscope (Olympus). For the detection of the virus encoded UL76 recruiting to DNA damage sites, MRC-5 cells were seeded on glass-bottomed culture dishes and infected with HCMV at an MOI of 1. At 48 h post-infection, infected cells (enlarged cell volume) were selected and irradiated. The immuno-staining protocol was the same as above.

For monitoring the expression and localization of PARP-1 and PARG in HCMV-infected cells, the MRC-5 cells were seeded on coverslips precoated with Matrigel (BD, 356234). Cells were infected with HCMV at an MOI of 0.1 and samples were collected at indicated time points. Coverslips were washed with PBS and fixed in cold methanol/acetone (1:1; −20 °C) for 15 min. After blocking in 10% FBS in PBS for 1 h, cells were co-stained with PARP-1 (rabbit, 1:200, Cell Signaling Technology, 9532) and UL44 (mouse, Santa Cruz, sc-69744, 1:250) or PARG (rabbit, Proteintech, 27808-1-AP, 1:100) and UL44 antibodies for 16 h at RT. Following washing with PBS for 5 times, the cells were incubated with FITC-conjugated anti-rabbit IgG (H + L) (1:500) and TRITC-conjugated anti-mouse IgG (H + L) (1:500) supplemented with DAPI (1:10,000) for 1 h.

### 2.10. RNA Isolation, cDNA Synthesis and Semi-Quantitative PCR

At the indicated times post-infection, the cells were washed with PBS and Trizol reagent was used for isolating RNA from the cells. First strand cDNA was then synthesized from 2 μg of RNA using HiScript^®^ III 1st Strand cDNA Synthesis Kit (Vazyme). Primer pairs for amplification of *GAPDH* were CGCTCTCTGCTCCTCCTGTT and CCATGGTGTCTGAGCGATGT and those for *PARP-1* were CCTAAAGGCTCAGAACGACC and CATCGCTCTTGAAGACCAGC, for *PARG* were TTCACTTCAGACGCTACCTC and GTCTCTCATCAATTCTGAGTCC, for *IFN-β* were CAGCAGTTCCAGAAGGAGGA and AGCCAGGAGGTTCTCAACAA, for *ISG 15* were TGTCGGTGTCAGAGCTGAAG and AGAGGTTCGTCGCATTTGTC. All sequences are given from 5′ to 3′ orientation.

### 2.11. Protein Expression and Purification

PARP-1 coding sequence (CDS) was subcloned to pET28a with an N-terminal hexahistidine tag designated His-PARP-1. Full length and truncate mutants (1–200, 1–250, 1–300) of the UL76 open-reading frame (ORF) were subcloned to pGEX4T-1 with an N-terminal GST tag. All of the proteins were expressed in the *Escherichia coli* strain BL21 (DE3). For PARP-1 expression, cells were growing in LB medium containing 100 µM ZnCl_2_ and induced with 200 µM isopropyl β-D-1-thiogalactopyranoside (IPTG) at 16 °C for 20 h. Cells were resuspended in lysis buffer (50 mM HEPES, pH8.0, 500 mM NaCl, 0.1% NP40, 1 mM phenylmethylsulfonyl fluoride) and then lysed using a cell disrupter (Avestin, Ottawa, ON, Canada). Cell debris was pelleted for 20 min at 12,000 rpm and the supernatant was incubated with NITA beads (ABclonal) at 4 °C for 6 h. After several washing steps with an increasing concentration of imidazole from 20–40 mM, PARP-1 was released from the beads with eluant buffer (50 mM HEPES, pH8.0, 150 mM NaCl, and 400 mM imidazole). For UL76 expression, the cells were growing in LB medium and induced with 1 mM TPTG at 28 °C for 4 h. Cells were resuspended in NETN100 buffer supplement with 1 mM PMSF. After disrupting and pelleting at 12,000 rpm for 20 min, the supernatant was carefully collected and incubated with Glutathione Affinity resins (ABclonal). The protein-bounded resins were washed with NETN100 buffer for 5 times and released with eluant buffer (20 mM Tris, pH8.0, 150 mM NaCl, 1 mM DTT, 50 mM reduced glutathione).

### 2.12. PAR Synthesis and Purification

PAR was synthesized according to Harald et al. [42]. Briefly, the reaction mixture containing 100 mM Tris–HCl pH 7.8, 10 mM MgCl_2_, 1 mM NAD^+^, 10 mM DTT, 60 μg/mL histone 2A, 50 μg/mL oligonucleotide (GGAATTCC) and 150 nM PARP-1 was incubated at 37 °C for 1 h. Ice-cold trichloro-acetic acid (TCA) was added to a final concentration of 20% (v/v) to stop the reaction and to precipitate PAR. PAR was pelleted by centrifugation at 16,000 *g* for 10 min. After being washed twice with ice-cold ethanol, PAR was detached from proteins by incubation with 0.5 M KOH/50 mM EDTA for 30 min at 37 °C. The pH was adjusted to 7.5–8.0 to stop the reaction. PAR was purified by phenol–chloroform–isoamyl alcohol extraction, which was followed by ethanol precipitation. Finally, PAR was dissolved in water and the concentration was determined by UV absorbance at 259 nm.

### 2.13. ADP-Ribosylation Assay In Vitro

Recombinant His-tagged human PARP-1 (100 nM) was mixed with an increasing amount (3–12 μM) of GST or GST-UL76 in the reaction buffer containing 50 mM Tris–HCl, pH 7.5, 7.5 mM MgCl_2_, 50 mM NaCl, 0.25 mM DTT, and 50 μg/mL oligonucleotide (GGAATTCC), in a total reaction volume of 40 μL. The recombinant proteins were mixed in the reaction buffer and incubated for 40 min at RT before the addition of NAD^+^ (200 μM) to the reaction, which proceeded for 10 min at 37 °C. The reaction was stopped by mixing with SDS sample buffer and boiling (5 min, 95 °C) before subsequent analysis by immunoblotting.

### 2.14. PAR Overlay Assay

Equal amounts of purified recombinant GST, GST-UL76 and individual truncate of UL76 were separated by SDS-PAGE and transferred onto a nitrocellulose membrane, which was subsequently blocked with TBS-T buffer (Tris-HCl pH 7.5, 150 mM NaCl, 0.05% Tween) supplemented with 5% milk. The PAR polymers were diluted with TBS-T to a final concentration of 100 nM. The membrane was incubated in PAR solution for 1 h at RT. The membrane was extensively washed with TBS-T containing 1 M NaCl and then probed with anti-PAR antibody and HRP-coupled secondary antibody.

### 2.15. Statistical Analyses

Graphs were generated with Prism GraphPad software (version 6.01; IBM). Data are expressed as averages ± standard deviations (SD). Densitometry quantification was performed with ImageJ Software. Student’s *t*-test was used for the comparison of two experimental groups, one-way ANOVA followed by Dunnett’s multiple test or two-way ANOVA followed by Tukey’s multiple test were conducted for multiple testing among the groups and statistical significance was set at a *p* value < 0.05.

## 3. Results

### 3.1. PARP-1 and Its Enzymatic Activity Were Required for Efficient HCMV Replication in Fibroblast

**Figure 1:**
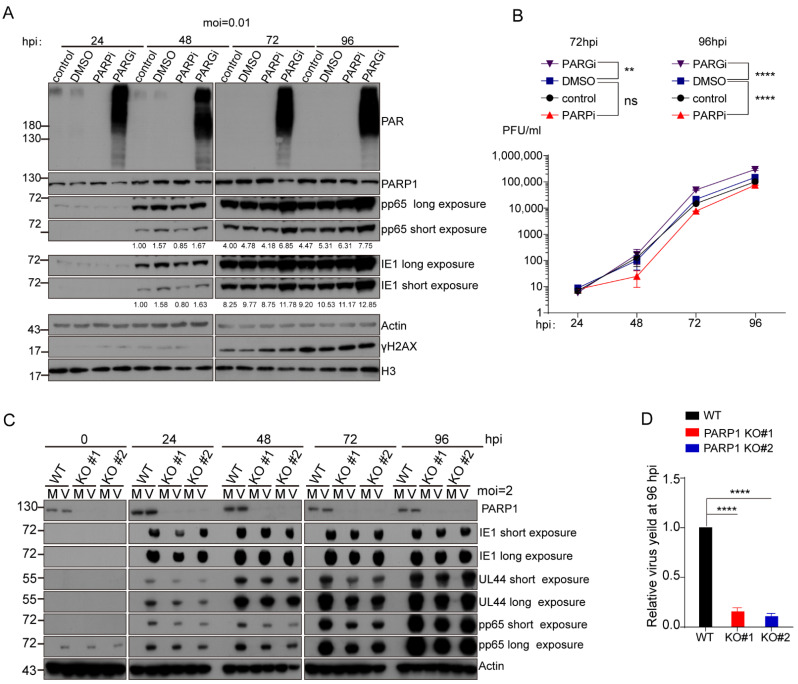
PARP-1 and its enzymatic activity were required for efficient HCMV replication in fibroblast. (**A**) MRC-5 cells were infected with HCMV at an MOI of 0.01. After 1 h adsorption, the virus was removed, and cells were washed with PBS. Fresh medium containing DMSO, 10 μM Olaparib or 10 μM PARG inhibitor was added to the cells and inhibitors were replenished every 24 h. Cells were collected every 24 h and cell lysates were analyzed by immunoblotting using indicated antibodies. IE1 and pp65 levels relative to those in control group at 48 h were quantified by Image J and values were presented below each blot. (**B**) Multistep growth curves of HCMV in PARPi or PARGi-treated MRC-5 cells. MRC-5 cells were infected and treated as panel (**A**). Released virus in the supernatant were collected and virus titers were determined by standard plaque-formation assay. The mean values are shown with bars denoting standard deviations for two independent experiments. ** *p* < 0.01, **** *p* < 0.0001. (**C**) PARP-1 depletion attenuates HCMV replication in immortalized Human Foreskin Fibroblasts (HFFs-hTERT). Wild type and two independent PARP-1 deficient HFFs-hTERT cell lines were mock infected (M) or infected with HCMV (V) at an MOI of 2. Cells were collected every 24 h and analyzed by immunoblotting with the indicated antibodies. (**D**) Progeny virus released to the culture at 96 hpi were collected and virus titers were analyzed by standard plaque formation assay. Values represent viral yields at 96 hpi and are expressed relative to WT HFFs-hTERT. Shown is the average of triplicate biological experiments (±1 SD), **** *p* < 0.0001.

We initially employed Olaparib (PARPi) and pDD00017273 (PARGi), small molecular inhibitors against PARP-1 and PARG, respectively, to investigate the role of PARP-1 and protein PARylation on HCMV infection. Treated MRC-5 cells with 10 μM PARPi, 10 μM PARGi or an equal volume of DMSO for 48 h do not affect cellular viability (Figure S1A). MRC-5 cells were infected with HCMV (AD169 strain) at a low multiplicity of infection (MOI = 0.01). After virus adsorption, the MRC-5 cells were refed with fresh medium containing 10 μM PARPi, 10 μM of PARGi or DMSO, respectively. Cells were collected every 24 h and the effects of PARPi or PARGi on HCMV replication was analyzed by Western blotting (WB) with indicated antibodies (Figure 1A). PARPi treatment abolished the PAR synthesis while PARGi treatment led to a significant accumulation of protein PARylation. This result confirmed that pharmacological inhibitors successfully changed the protein PARylation levels in HCMV-infected cells. The expression of the HCMV immediate early gene (IE1) and early/late gene (pp65) were compromised in PARPi-treated cells. Interestingly, when the PARG activity was inhibited, the expression of these two viral genes were increased. Viral titers in the supernatant were measured with standard plaque formation assay (Figure 1B). Viral yields decreased to about 50% of the control group in the PARPi treatment group at 96 h post-infection (hpi). PARGi treatment doubled viral yield at 96 hpi. Furthermore, we also examined the effect of PARPi or PARGi on HCMV replication when the MRC-5 cells were infected with HCMV at a high MOI (MOI = 1). The inhibitory effect of PARPi and promotive effect of PARGi on HCMV replication also existed when the cells were infected with a high MOI (Figure S1B,C). Without extra treatment, the protein PARylation levels decreased at late phase (72–96 hpi) of infection which indicated that HCMV infection actively manipulated protein PARylation levels in infected cells.

To further determine the role of PARP-1 on HCMV replication, human telomerase reverse transcriptase (hTERT)-immortalized Human Foreskin Fibroblasts (HFFs-hTERT) were employed to construct PARP-1 knockout cell lines. The wild-type or PARP-1 knockout HFFs-hTERT were infected or mock infected with HCMV at an MOI of 2. The samples were collected every 24 h and the effect of PARP-1 depletion on HCMV replication was analyzed by WB and standard plaque-formation assay (Figure 1C,D). In the PARP-1 knockout cells, viral genes (IE1, UL44 and pp65) expression was attenuated and progeny virus production decreased to less than 20% of WT HFFs-hTERT at 96 hpi, indicating PARP-1 depletion caused a severe reduction in the viral yield.

### 3.2. HCMV Infection Activated PARP-1 and Induced Its Translocation from Nucleus to Cytoplasm

**Figure 2:**
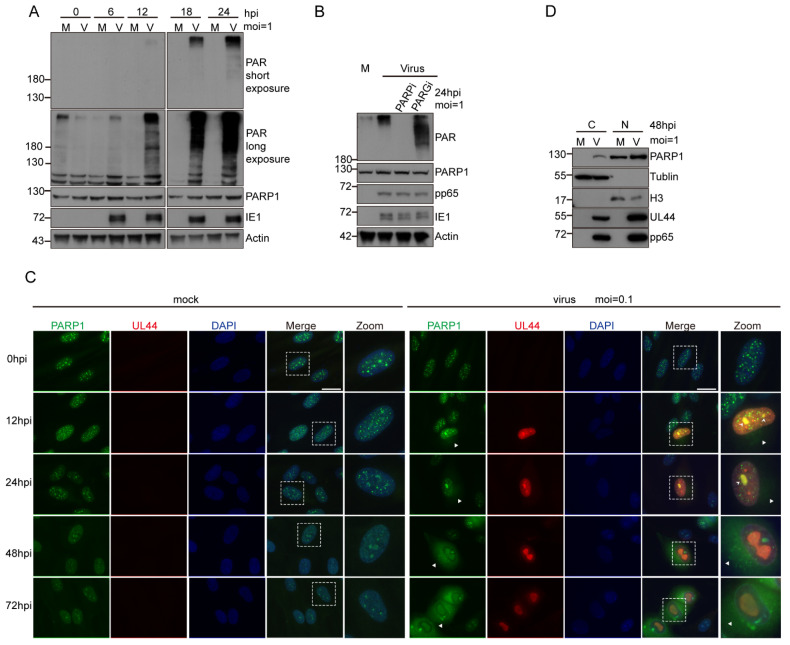
HCMV infection induced increase in protein PARylation and cytosolic translocation of PARP-1. (**A**) Analysis of protein PARylation levels at indicated times after HCMV infection. MRC-5 cells were mock infected (M) or infected (V) with HCMV (MOI = 1) and harvested at various times post-infection. Protein PARylation was detected by WB with PAR antibody. IE1 served as a marker for HCMV infection. (**B**) The increase in protein PARylation levels was due to PARP-1 activation. MRC-5 cells were infected with HCMV at an MOI of 1. After virus adsorption, small molecule inhibitor to PARP-1 (Olaparib 10 µM) or PARG (PDD 00017273 10 µM) was added to the medium. Cells were harvested at 24 hpi and protein PARylation levels were analyzed. (**C**,**D**) HCMV infection induced the cytosolic translocation of PARP-1 in MRC-5 cells. (**C**) MRC-5 cells were infected with HCMV at an MOI of 0.1 and fixed with cold methanol/acetone (1:1) at various times. Expression profiles and localization of PARP-1 and HCMV UL44 over the course of HCMV infection were examined by immunofluorescence assay. Triangles indicated the repositioned PARP-1 in cytoplasm. Arrowheads indicated the colocalization between PARP-1 and UL44. Zoomed pictures were also provided to show the cytoplasmic translocation of PARP-1. Scale bar, 20 μm. (**D**) Immunoblotting of indicated proteins in the cytoplasmic and nuclear fractions of MRC-5 cells infected (V) or mock infected (M) with HCMV at an MOI of 1 for 48 h. “C” refers to cytoplasmic and “N” refers to nuclear.

It was reported that PARP-1 was activated in HSV-1- [33] and HBV- infected cells [43]. However, the effect of HCMV infection on the activation of PARP-1 is still unknown. We first examined whether HCMV infection activates PARP-1 in fibroblasts. MRC5 cells were infected with HCMV at an MOI of 1, and a detectable accumulation of protein PARylation was evident as early as 12 hpi (Figure 2A). To define whether the accumulation of PARylated proteins in HCMV-infected cells was due to PARP-1 activation, Olaparib, a small molecular inhibitor specific to PARP-1, was employed. Following virus adsorption for 1 h, Olaparib was applied in parallel with virus infection. At 24 hpi, the cells were harvested and the protein PARylation levels were analyzed by WB. Olaparib treatment totally inhibited the increase in protein PARylation upon HCMV infection. On the contrary, when the PARG activity was blocked by PDD00017273, protein PARylation levels were further increased (Figure 2B). These results indicated that HCMV infection led to PARP-1 activation.

Immunofluorescence was employed to detect the distribution of PARP-1 in HCMV-infected cells. MRC-5 cells were infected or mock infected with HCMV at an MOI of 0.1. Cells were fixed at indicated time points post infection and stained with PARP-1 antibody. HCMV UL44 was co-stained to indicate HCMV-positive cells. PARP-1 remained in the nucleus in the mock infected cells. However, HCMV infection induced the translocation of PARP-1 from the nucleus to cytoplasm (Figure 2C). The reposition of PARP-1 was evident as early as 12 hpi. Cell fraction followed by WB analysis also demonstrated the translocation of PARP-1 in HCMV-infected cells (Figure 2D). If not all, some of the colocalization of PARP-1 and the viral replication compartments (vRCs, as visualized by UL44) were observed in HCMV-infected cells when fixed with methanol/acetone (Figure 2C), suggesting that PARP-1 may be involved in the viral DNA replication process.

### 3.3. PARG Was Upregulated during HCMV Infection, and This Upregulation Was Independent of Viral DNA Replication

**Figure 3:**
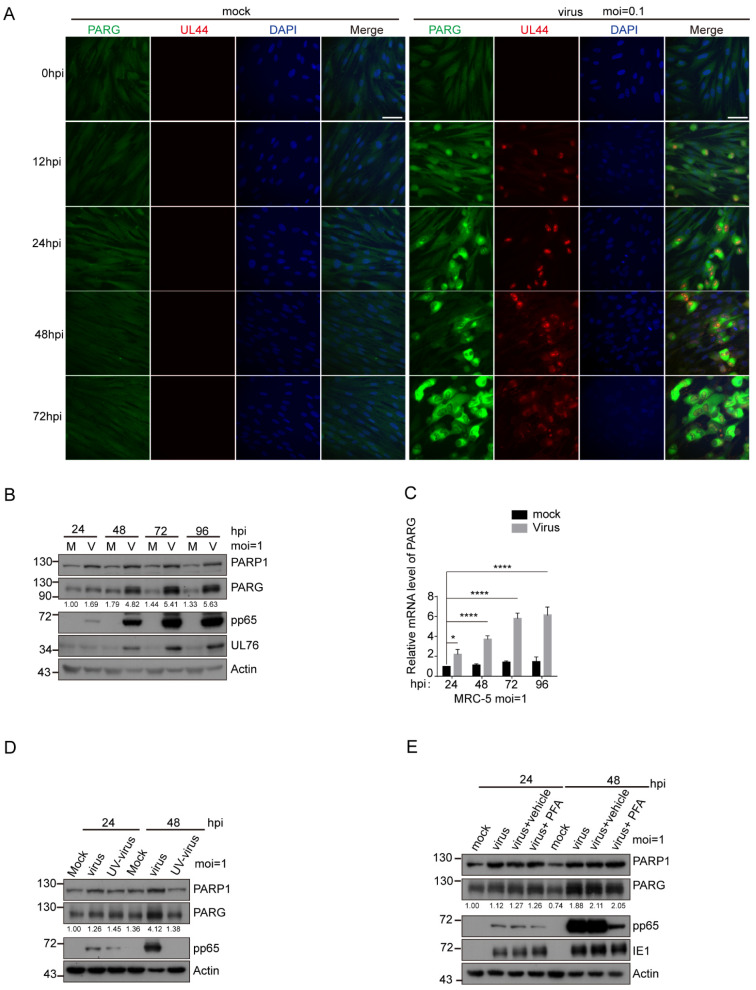
PARG is continuously upregulated over the course of HCMV infection. (**A**) The expression profiles of PARG and HCMV UL44 in HCMV-infected MRC-5 cells were analyzed by IFA. MRC-5 cells were infected or mock infected with HCMV at an MOI of 0.1. At indicated times post infection, cells were fixed with methanol/acetone (1:1) and stained with PARG and HCMV UL44 antibody. Scale bar, 50 μm. (**B**) HCMV infection upregulates PARG continuously. Expression of PARG and two viral proteins were detected at indicated time points after HCMV infection. MRC-5 cells were infected with HCMV at an MOI of 1. Infected (V) or mock-infected (M) cells were harvested at a 24 h interval for four days. The protein levels of PARP-1, PARG were analyzed by Western blot. PARG levels relative to those in mock-infected cells at 24 h was quantified by ImageJ and values were presented below each PARG blot. (**C**) HCMV infection increased the transcription of PARG. Relative mRNA levels of PARG were analyzed by RT-qPCR. Data were normalized to the levels in mock-infected cells at 24 hpi to provide fold changes after HCMV infection. Data were analyzed by one-way ANOVA. * *p* < 0.05, **** *p* < 0.0001. (**D**) The upregulation of PARG required de novo expression of HCMV genes. MRC-5 cells were infected with HCMV or UV-inactivated (5 J/cm^2^) virus, and the expression of PARP-1 and PARG were analyzed at 24 and 48 hpi. Fold changes in PARG expression were determined by ImageJ and values were indicated below each PARG blot. (**E**) The upregulation of PARG in HCMV-infected fibroblast was independent of viral DNA replication. MRC-5 cells were infected with HCMV at an MOI of 1, and in parallel with virus inoculation, 250 μM phosphonoformic acid (PFA) was added to the infected cells as indicated. Cells were harvested at 24 and 48 hpi, and whole cell lysates were analyzed by WB with indicated antibodies. Fold changes in PARG expression was determined by ImageJ and values were indicated below each PARG blot.

PARP-1 and PARG functioned as the main “writer” and “eraser” for protein PARylation in mammal cells. As HCMV infection activated PARP-1 and induced the reposition of PARP-1, we want to know whether HCMV infection also affected the expression of PARG. MRC-5 cells were infected or mock infected with HCMV at an MOI of 0.1. At indicated time points post infection, the cells were fixed and stained with PARG and HCMV UL44 antibodies. As mentioned before, human PARG is encoded by a single gene, and alternative splices produced three major PARG isoforms that target different cell compartments. The full-length 111 KD isoform targets the nucleus and the two short isoforms (102 KD and 99 KD) are cytoplasmic proteins [26]. The PARG antibody used in this research recognized three of these isoforms and was more efficient in detecting the two cytosolic isoforms. We found that PARG was upregulated in HCMV-infected cells (Figure 3A). To distinguish which isoform was upregulated upon HCMV infection, we also performed WB analysis. MRC-5 cells were infected or mock infected with HCMV at an MOI of 1. It is clearly shown that all of the three isoforms were upregulated in HCMV-infected cells (Figure 3B). We examined the mRNA level of *PARG* by RT-qPCR and found that *PARG* mRNA continuously increased after HCMV infection (Figure 3C). Thus, we demonstrated that HCMV infection induced the upregulation of PARG.

To explore the underlying mechanisms that regulate PARG expression after HCMV infection, we introduced UV-inactivated HCMV and phosphonoformic acid (PFA). The UV-inactivated HCMV was unable to induce the upregulation of PARG (Figure 3D). That means the upregulation of PARG was not caused by the incoming viral components, such as the tegument or the capsid. HCMV gene expression occurs in a cascade fashion with chronological phases termed immediate early (IE), early (E) and late (L). To further confine the viral genes that are responsible for inducing upregulation of PARG during HCMV infection, we used the viral DNA synthesis inhibitor phosphonoformic acid (PFA). PFA (250 ug/mL) was applied to the cell in parallel with HCMV infection and the expression profile of PARG was analyzed by WB. The results showed that the increase in PARG was not affected by inhibition of viral DNA replication through PFA treatment (Figure 3E, compared lane 2/3 vs. 4, lane 6/7 vs. lane 8). PFA treatment significantly decreased the expression of HCMV pp65. Taken together, we concluded that the HCMV-mediated elevation of PARG protein levels is independent of viral DNA replication.

### 3.4. HCMV UL76 Bound to the BRCT Domain of PARP-1

**Figure 4:**
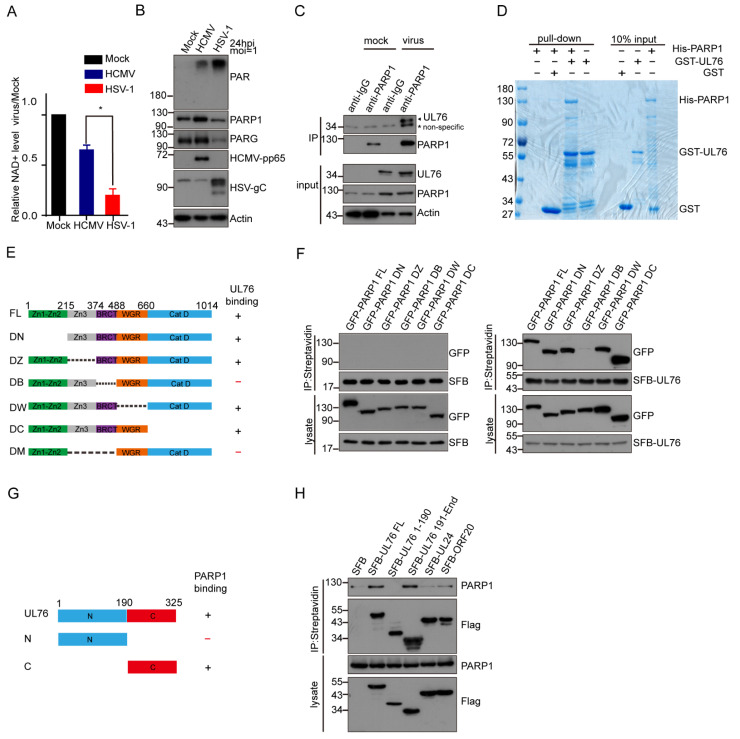
HCMV UL76 binds to the BRCT domain of PARP-1 through its C terminal. (**A**) NAD^+^ levels declined to varying degrees upon HSV-1 and HCMV infection. Fibroblasts were infected with HSV-1 (F strain) or HCMV (AD169 strain) at an MOI of 1. Cells were collected at 24 hpi by trypsin digestion and lysed in a NAD^+^ extraction buffer. NAD^+^ levels in these cells were normalized to total protein and compared to mock-infected cells. Values are averages of triplicate experiments (±1 SD), * *p* < 0.05. (**B**) Protein PARylation and the protein levels of PARP-1 and PARG were compared between HSV-1- and HCMV-infected cells. MRC-5 cells were infected with HSV-1 or HCMV at an MOI of 1. Pp65 of HCMV and glycoprotein C of HSV-1 served as infection markers for each virus. The whole cell lysates were analyzed by indicated antibodies. (**C**) HCMV tegument protein UL76 was a PARP-1 binding protein. MRC-5 cells were infected or mock infected with HCMV at an MOI of 1 for 96 h. Cells were harvested and lysed in NETN 420 in the presence of protease inhibitors. Cell lysates were prepared and subjected to immunoprecipitation with PARP-1 antibody or control IgG. The presence of PARP-1 and UL76 in the immune complexes was determined by Western blot analysis. * indicates nonspecific band. (**D**) UL76 directly interacts with PARP-1 in vitro. Purified His-PARP-1 was incubated with equal amount of GST or GST-UL76. Samples were subjected to GST pull-down, and bound complexes were analyzed by SDS-PAGE. (**E**) Schematic depiction of PARP-1 and its deletion mutants. Results from domain mapping experiments indicated that UL76 binds to the BRCT domain of PARP-1. (**F**) BRCT domain of PARP-1 was responsible for the association between UL76 and PARP-1. SFB vector or SFP-UL76 was co-transfected with GFP-tagged PARP-1 WT or each deletion mutant. UL76 did not bind with the PARP-1 BRCT domain deletion mutant. (**G**) Schematic depiction of HCMV UL76. Results from domain mapping experiments indicated that PARP-1 binds to the C terminal of UL76. (**H**) HEK293T cells were transfected with a construct for SFB vector, SFB-tagged UL76, UL76 truncates or UL76 homologs in HSV-1 and KSHV, respectively. Cell lysates were subjected to SFB immunoprecipitation, and the presence of PARP-1 was analyzed by immunoblotting.

The previous reports have demonstrated that NAD^+^ levels dropped dramatically in HSV-1-infected cells (by a factor of about 10), but little decline was evident after HCMV infection [44]. We compared the NAD^+^ levels in HSV-1 and HCMV-infected fibroblasts. MRC-5 cells were infected with HCMV (AD169 strain) or HSV-1 (F strain) at an MOI of 1. At 24 hpi, infected or mock-infected cells were collected and lysed in NAD^+^ extraction buffer. Consistent with the previous study [44], the NAD^+^ levels dropped to about 20% in HSV-1-infected cells compared with the mock-infected group (Figure 4A). The decline in the NAD^+^ levels in HCMV-infected cells was moderate and about 60% of NAD^+^ was kept in HCMV-infected cells. In addition, we assessed the PARylation levels in HCMV- or HSV-1-infected MRC-5 cells. HSV-1 infection led to higher protein PARylation than HCMV infection (Figure 4B). In contrast to HCMV infection, PARG was downregulated in HSV-1-infected cells which was consistent with the previous study [33].

HCMV infection only caused a moderate decline in cell NAD^+^ levels, implying that PARP-1 activation may be tightly regulated to prevent its over-activation and dramatic NAD^+^ decrease after HCMV infection. Viruses, such as HBV [31], adenovirus [32], EBV [34,45] and KSHV [37,38,39,40] have been reported to encode PARP-1 partners. We speculated that PARP-1 activation could be regulated by HCMV-encoded proteins to maintain relatively stable NAD^+^ levels.

For the purpose of identifying PARP-1 partners encoded by the HCMV genome, we first searched for candidates from published papers. Nobre and colleagues performed a mass spectrometry-based interactome analysis and unveiled a detailed interactome of HCMV-encoded proteins [46], among which UL76 of HCMV was identified as a partner of PARP-1. The HCMV UL76 protein is virion-associated and expressed with true late kinetics [13]. To determine whether UL76 interacts with PARP-1, we first raised a rabbit polyclonal antibody against HCMV UL76. The specificity of this antibody was confirmed by WB in UL76-overexpressed 293T cells (Figure S2A) and in HCMV-infected MRC-5 cells (Figure S2B). Meanwhile, when viral DNA replication was inhibited by PFA, the signals detected with this antibody were significantly suppressed (Figure S2C), which was consistent with the fact that UL76 was expressed with true late kinetics and its expression depends on viral DNA replication [13].

To investigate whether PARP-1 and UL76 form a complex in HCMV-infected cells, the cell lysates from HCMV-infected MRC-5 cells expressing UL76 endogenously were immunoprecipitated with control IgG or PARP-1 antibody. UL76 was detected in a protein complex immunoprecipitated by PARP-1 antibody but not the control IgG (Figure 4C). Moreover, no binding between PARP1 and HCMV pp65 was detected in HCMV-infected MRC-5 cells (Figure S2D). Next, we performed an in vitro GST pull-down assay using GST-UL76 and His-PARP-1 (Figure 4D), which also indicated that GST-UL76 was directly bound with His-PARP-1.

PARP-1 contains multiple domains, including the N terminal zinc finger domain (Znf1-Znf2, 1–215 aa termed N domain), the central auto-modification domain (216–488 aa, M domain), a WGR domain (489–660 aa, W domain) and a C-terminal catalytic domain (661–1014 aa, C domain). The auto-modification domain was further divided into a third zinc finger fold (Znf3, 216–374 aa, Z domain) and a BRCT fold (375–488 aa, B domain) (Figure 4E and Figure S3A). The BRCT fold is present in several DNA repair factors and is frequently found to mediate protein–protein interactions [47]. To further characterize the interaction between PARP-1 and UL76, several SFB-tagged truncation and deletion mutants of PARP-1 were constructed and their binding ability with UL76 was tested. We found that UL76 was bound to the full length (FL) and auto-modification domain (M) of PARP-1 (Figure S3B). Deletion of the auto-modification domain (termed DM) disrupted the binding ability of PARP-1 with UL76 (Figure S3C). These results indicated that the M domain of PARP-1 was responsible for binding with UL76. Furthermore, we found that the BRCT domain deletion mutant of PARP-1 (PARP-1 DB) failed to associate with UL76 (Figure 4F). Collectively, we concluded that UL76 is bound to the BRCT domain of PARP-1. We also examined the association between UL76 and PARP-1 when PARP-1 was activated or inhibited (Figure S3D). It seems that the activation or inhibition of PARP-1 activity does not affect the association between UL76 and PARP-1.

UL76 was divided into two regions based on the sequence alignments of Herpes_UL24 family members [14], including the conserved N-terminal (amino acids 1 to 190) and the variable C-terminal (amino acids 191 to 325) (Figure 4G). To determine the region responsible for UL76 binding to PARP-1, SFB-tagged UL76, UL76 N or C terminus, or the homologs of UL76 (UL24 of HSV-1 and ORF20 of KSHV) were transfected to HEK293T cells, respectively. Immunoprecipitation experiments indicated that only UL76 but not UL24 or ORF20 could bind with endogenous PARP-1, and the C-terminus of UL76 was responsible for its interaction with PARP-1 (Figure 4H).

### 3.5. HCMV UL76 Inhibited PARP-1 Activity Both In Vivo and In Vitro

**Figure 5:**
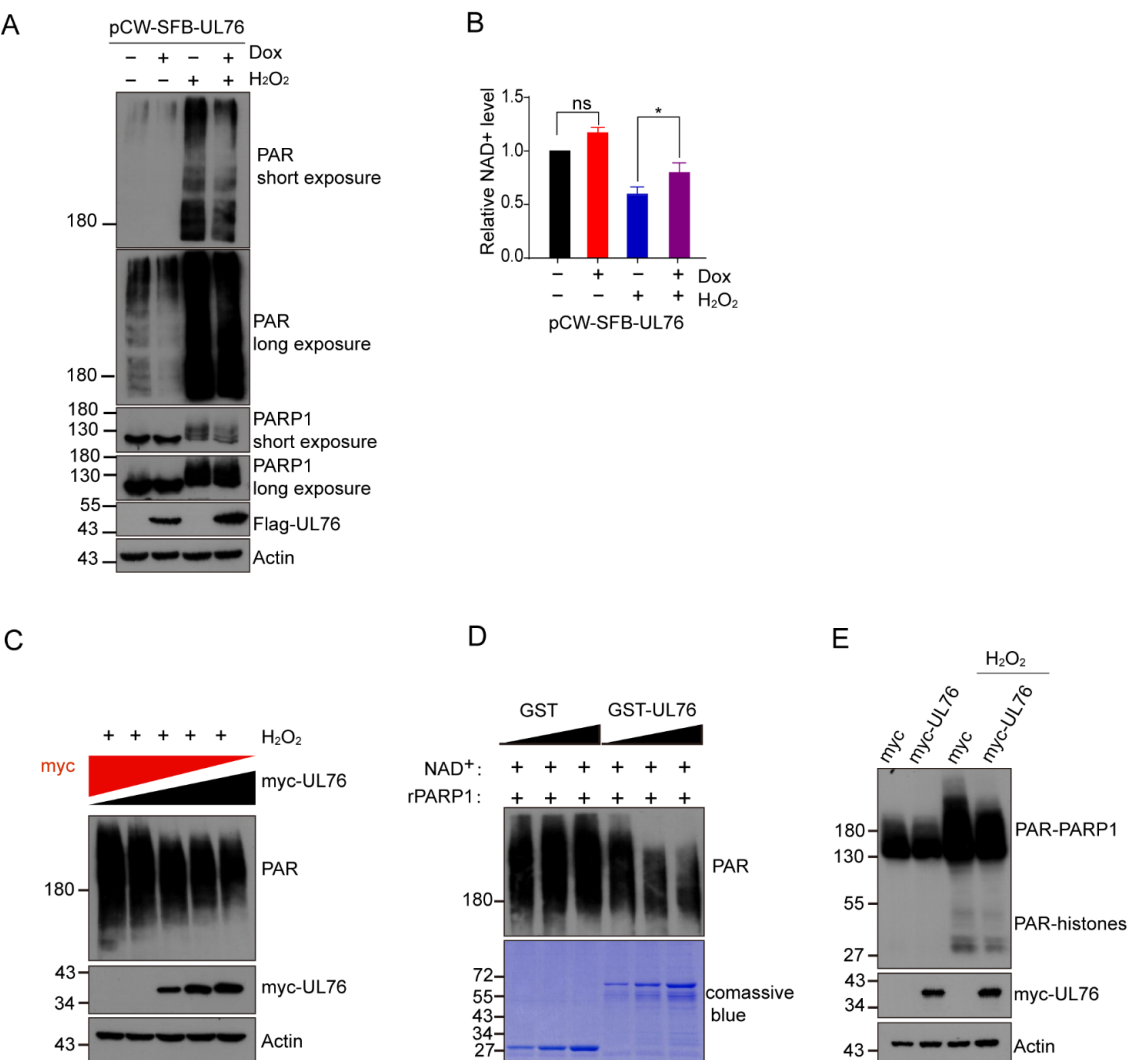
UL76 inhibited protein PARylation. (**A**) UL76 inhibited the overactivation of PARP-1 induced by H_2_O_2_ treatment in the UL76 inducible RPE cellular system. Whereby doxycycline (Dox) addition led to the inducible expression of SFB-tagged UL76 in RPE cells. These cells were induced with or without Dox (1µg/mL) for 24 h, followed by incubation with 200 µM H_2_O_2_ for 5 min. Whole cell lysates were analyzed with the indicated antibodies. (**B**) UL76 mitigated the NAD^+^ depletion induced by H_2_O_2_ treatment in UL76 inducible cells. UL76 inducible RPE cells were induced with Dox (1 µg/mL) for 24 h or left untreated. Cells were then challenged with 500 µM H_2_O_2_ for 10 min, and NAD^+^ levels were measured. Each treatment is the average of triplicate biological experiments (±1 SD). The *p*-values were calculated using two-tailed Student’s *t*-tests. ns, not significant; *, *p* < 0.05. (**C**) UL76 inhibited H_2_O_2_-induced protein PARylation in a dose-dependent manner. HEK293T cells were co-transfected with a plasmid encoding myc-UL76 and the empty vector, increasing amounts of plasmid encoding myc-UL76 and correspondingly decreasing amounts of the empty vector. Forty-eight hours post-transfection, the cells were treated with 1 mM H_2_O_2_ for 5 min and protein PARylation was detected immediately with PAR antibody. Western blot analysis showed that protein PARylation decreased continuously following increased overexpression of myc-UL76. (**D**) UL76 directly inhibited PARP-1 activity in vitro. Recombinant His-PARP-1 was incubated with increasing amount of purified GST or GST-UL76 for 30 min in the reaction buffer. A total of 200 μM NAD^+^ was added to initiate the PARylation reaction and maintained for 10 min at 37 °C. Reaction products were separated by SDS-PAGE and analyzed by immunoblotting with PAR antibody. (**E**) UL76 inhibited both PARP-1 auto-modification and histone PARylation. Empty vector or myc-tagged UL76 were transfected to HEK293T cells. At 48 h post-transfection, cells were treated with H_2_O_2_ (1 mM) for 5 min, and whole cell lysates were analyzed by immunoblotting with the indicated antibodies. ADP-ribosylated PARP-1 (PAR-PARP-1) and histones (PAR-Histones) are indicated.

To examine whether UL76 affects PARP-1 enzymatic activity, we constructed Tet-On inducible SFB-UL76 in the RPE cells (Figure S4A,B). Cells were induced with or without doxycycline (Dox, 1 μg/mL) for 24 h and counterstained with Flag and PARP-1 antibodies. The results from indirect immunofluorescence assay and Western blot confirmed this cell system functioned correctly (Figure S4A,B). Consistent with the previous study [14], UL76 was detected as nuclear aggresomes in an indirect immunofluorescence assay and with a predicted mass weight of 50 Kd when fused with SFB tag in WB. Colocalization of SFB-UL76 and PARP-1 was evident in the Dox-induced cells (Figure S4A). We found that the Dox-induced overexpression of SFB-UL76 decreased the endogenous protein PARylation (Figure 5A). In contrast, Dox treatment in normal RPE cells did not have a noticeable effect on protein PARylation (Figure S4C). As H_2_O_2_ treatment induced PARP-1 overactivation [48], the effect of UL76 on H_2_O_2_-induced protein PARylation was tested. UL76 prevented the H_2_O_2_-induced accumulation of protein PARylation (Figure 5A). Next, we examined the endogenous NAD^+^ level after SFB-UL76 induction. The H_2_O_2_ treatment obviously led to a decrease in cellular NAD^+^ level, which could be partially rescued by SFB-UL76 overexpression (Figure 5B). Meanwhile, the decrease in PAR positively correlated with the amount of UL76 overexpression (Figure 5C) in H_2_O_2_-treated cells. These results clearly indicated that UL76 affected the endogenous PARP-1-mediated protein PARylation.

We also examined the effect of UL76 on PARP-1 activity through in vitro assay and found that GST-UL76, but not GST protein, inhibited the auto-modification of His-PARP-1 (Figure 5D). Two major targets of PARP-1 catalytic activity are PARP-1 itself and histones, which are both clearly evident by the immunoblotting of whole-cell extracts with anti-poly (ADP-ribose) antibodies [48]. The molecular weight of PARylated histones and PARylated PARP-1 ranged from 17 KD to more than 200 KD, gradient SDS-PAGE (from 4–12%) was used to separate these ADP-ribosylated proteins. To determine whether the PARylation of histones could also be affected by UL76, we examined the PARylation of histones after H_2_O_2_ treatment. UL76 overexpression led to a mild decrease in histone PARylation levels (Figure 5E), indicating that UL76 could also affect the modification of other substrates by PARP-1. These results suggested that UL76 could inhibit PARP-1 activity and alleviate H_2_O_2_-induced NAD^+^ decrease, which confirmed our speculation that HCMV encoded genes could counteract the hyper-activation of PARP-1 and the dramatic reduction in the cellular NAD^+^ pool.

### 3.6. PAR Dependent Recruitment of HCMV UL76 to DNA Damage Sites

**Figure 6:**
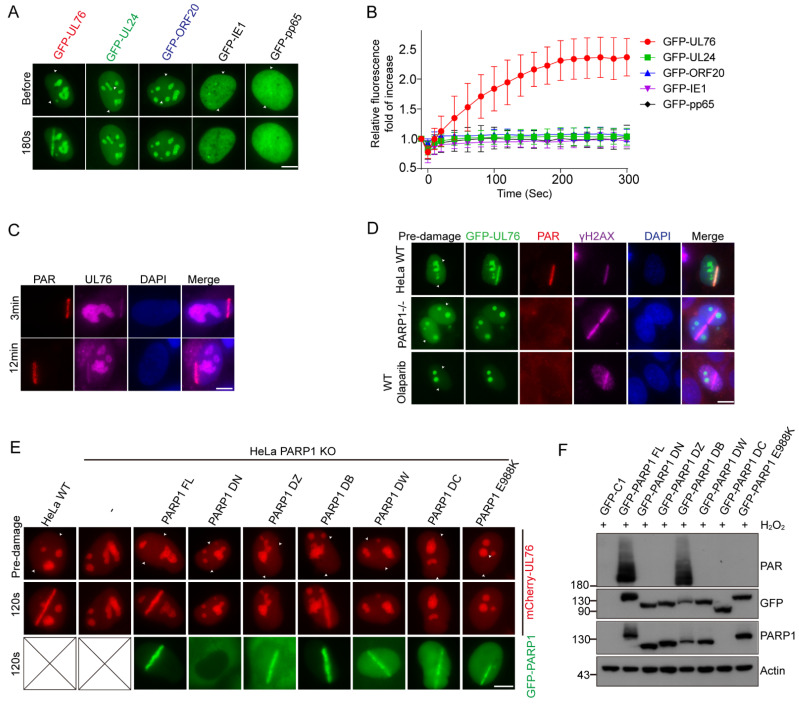
PAR-dependent recruitment of HCMV UL76 to DNA damage sites. (**A**) Several Herpesvirus genes were tested for their ability to accumulate at DNA lesions induced by laser micro-irradiation. Members of the Herpesvirus_UL24 family in HCMV (UL76), HSV-1 (UL24), KSHV (ORF20) and HCMV immediate early gene IE1, low matrix protein pp65 were transfected to U2OS cells with a GFP tag on their N terminus. Cells were subjected to laser irradiation, and live cell images were captured at indicated time points. Paired arrowheads indicated the orientation of the laser track. Scale bar, 10 μm. (**B**) Kinetics of the recruitment of UL76 and other tested proteins to DNA lesions were measured over 5 min post irradiation. The recruitment of these fluorescent proteins was quantified by the ratio of GFP intensity at the laser track normalized to GFP intensity at the same region pre-damage. The data are shown as mean ± SD, n = 14. (**C**) The recruitment of endogenous UL76 to DNA lesions in HCMV-infected cells. MRC-5 cells were infected with HCMV at an MOI of 1. Forty-eight hours post-infection, HCMV infected cells were selected for laser irradiation. Cells were then fixed and immunostained with indicated antibodies. Scale bar, 10 μm. (**D**) Recruitment of UL76 to DNA lesions depends on PAR synthesis. WT and PARP-1 KO HeLa cells were transfected with GFP-UL76 and subjected to micro-irradiation. Cells were fixed and stained with PAR and γH2AX antibodies. In the Olaparib treatment panel, cells were pretreated with 10 μM Olaparib for 30 min before laser irradiation. Paired arrowheads indicated the orientation of the laser track. Scale bar, 10 μm. (**E**) PARP-1 knockout HeLa cells were co-transfected with mCherry-UL76 and GFP-PARP-1 full length or the indicated deletion mutant. Cells were subjected to laser irradiation at 24 h post-transfection. The recruitment of mCherry-UL76 and GFP-PARP-1 was monitored simultaneously. Paired arrowheads indicated the orientation of the laser track. Scale bar, 10 μm. (**F**) Protein PARylation in PARP-1 knockout HEK293T cells rescued with PARP-1 WT, or indicated mutants was shown. PARP-1 knockout HEK293T cells were transfected with GFP-tagged PARP-1 WT, its enzymatic death mutant (E988K) or each deletion mutant. H_2_O_2_-induced protein PARylation was analyzed by PAR antibody.

The most significant feature of UL76 is that it contains many positive charged residues with a putative pI of 11.6 [13,14]. As the PAR chains are comprised of negative-charged ADP-ribose, we speculated whether UL76 directly interacts with the PAR chains. To study the association between UL76 and PAR, we employed laser micro-irradiation assay. Laser micro-irradiation leads to the immediate recruitment of PARP-1 on DNA damage sites and produces a large amount of PAR polymers on these sites [49,50,51]. Based on these aspects, PAR-associated proteins could accumulate at micro-irradiation induced strips through PAR binding. Using this method, we can explore whether UL76 could bind with PAR. We examined the localization of UL76 and its homologs in HSV-1 or KSHV using a micro-irradiation assay and found that only UL76 accumulated at the DNA damage strips (Figure 6A). We also examined the localization of GFP-IE1 and GFP-pp65 using a micro-irradiation assay and found that these proteins could not accumulate at DNA damage sites. Meanwhile, the dynamic of the recruitment of these GFP-tagged proteins was monitored and images were captured at 20 s intervals by the live-cell imaging method. The recruitment of UL76 at the DNA damage sites occurred as early as 30 s and peaked at 5 min post micro-irradiation (Figure 6B).

To determine whether endogenous UL76 could accumulate at the micro-irradiation strips, we treated HCMV-infected MRC-5 cells with laser micro-irradiation and endogenous UL76 was immunostained with UL76 antibody. The laser micro-irradiation assay was conducted in MRC-5 cells 48 h after HCMV infection. These irradiated cells were fixed and immunostained with PAR and UL76 antibodies. We found that endogenous UL76 clearly co-localized with PAR at the DNA damage strips (Figure 6C).

As PARP-1 is responsible for most of the PAR formation at the DNA damage strips, we studied the role of PARP-1 on the accumulation of UL76 at DNA damage sites. The recruitment of UL76 at the DNA damage strips disappeared in the PARP-1 KO HeLa cells (Figure 6D) and Olaparib treatment also blocked the accumulation of UL76 at the DNA damage sites (Figure 6D), suggesting that the recruitment of UL76 on DNA damage strips was dependent on PARP-1 activity but not the binding capacity of UL76 with PARP-1 protein. PAR and γH2AX were immunostained to indicate the DNA damage sites.

To further exclude the possibility that the direct binding between UL76 and PARP-1 contributed to the accumulation of UL76 on the DNA damage sites, several GFP-tagged PARP-1 mutants were introduced to PARP-1 deficient HeLa cells, and their ability to restore the recruitment of UL76 to the DNA damage sites was studied (Figure 6E). Interestingly, apart from the WT PARP-1, the DB mutant of PARP-1, which was unable to bind with UL76 (Figure 4F), also restored the recruitment of UL76. Meanwhile, the DN, DZ, DW, DC and enzymatic death mutant (E988K) of PARP-1 were unable to restore this phenomenon. The ability of PAR synthesis upon H_2_O_2_ treatment of these mutants in PARP-1 KO cells was checked by WB (Figure 6F). Consistent with previous research [52], the WT and DB mutant of PARP-1 kept the PAR synthesis ability upon H_2_O_2_ treatment. Collectively, these results demonstrated that the accumulation of UL76 at DNA damage sites did not depend on its binding ability with PARP-1 but depended on PAR synthesis.

### 3.7. HCMV UL76 Bound to PAR via Its RG/RGG Motif in the C Terminal

**Figure 7:**
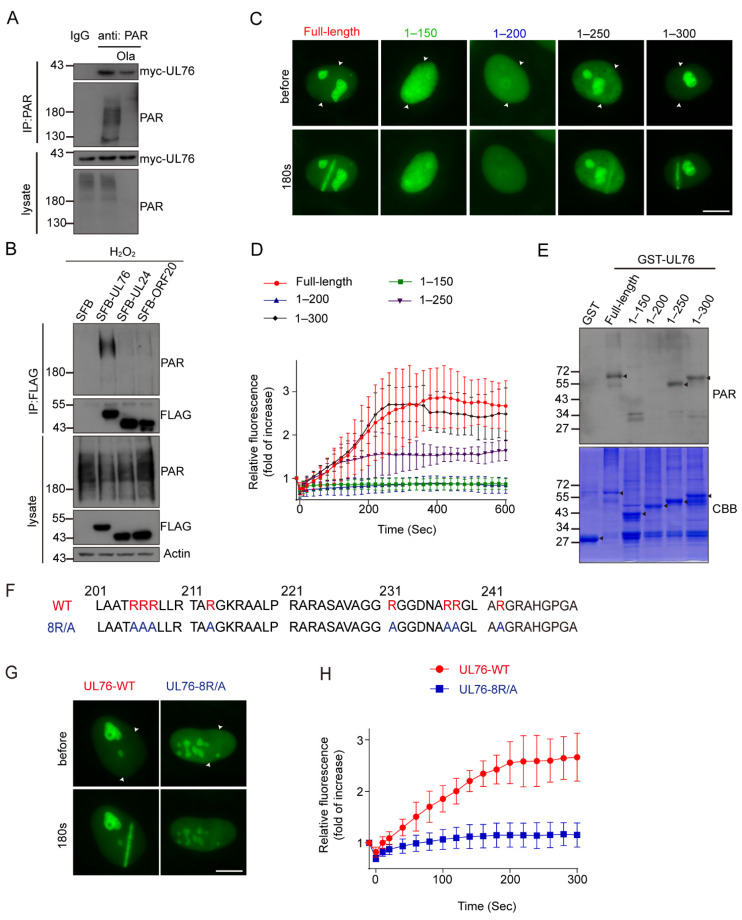
UL76 directly bound to PAR via RG/RGG motif. (**A**) Myc-UL76 was transfected to 293T cells. The cells were harvested, and the whole cell extracts were subjected to immunoprecipitation with PAR antibody or control IgG. The presence of PAR and UL76 in the immune complexes was determined by Western blot with indicated antibodies. Olaparib treatment inhibited the production of PAR and decreased the amount of UL76 immunoprecipitated by PAR antibody. (**B**) SFB-tagged UL76 or its analogs were transfected to HEK293T cells for 48 h. Cell lysates were subjected to immunoprecipitation assay using Flag antibody. Only UL76 could pull down endogenous PAR chains. (**C**) Mapping of the decisive region in UL76 for its ability to accumulate at DNA damage sites. Different truncates of UL76 were transfected to HeLa cells. GFP-positive cells were irradiated by laser micro-irradiation. Paired arrowheads indicated the orientation of the laser track. Scale bar, 10 μm. (**D**) Kinetics of the recruitment of these UL76 truncates to DNA lesions were quantified for 10 min in panel C. Fluorescent signals at laser tracks were recorded at indicated times and normalized to pre-damage. The data are shown as mean ± SD, n = 5. (**E**) The PAR overlay assay showed that 200–250 aa of UL76 was responsible for its PAR binding. Different truncates of UL76 were expressed in *E. coli* and purified. These proteins were separated by SDS-PAGE and transferred to nitrocellulose membrane. Membrane was blocked and then incubated with 100 nM PAR. The binding of PAR to these truncates was probed with PAR antibody. (**F**) Putative RG/RGG repeats in 200–250 aa of UL76. Key arginines in these repeats are labeled with red. In 8R/A mutant, R205, R206, R207, R213, R231, R237, R238 and R242 were mutated to Alanine (A). (**G**) WT or 8R/A mutant of UL76 were transfected to HeLa cells. The recruitment of UL76 or 8R/A mutant to DNA damage sites was determined by laser micro-irradiation. Paired arrowheads indicated the orientation of the laser track. Scale bar, 10 μm. (**H**) Kinetics of the recruitment of WT and 8R/A mutant of UL76 to DNA lesions were measured for 5 min. The fluorescent intensity was normalized to pre-damage. The data are shown as mean ± SD, n = 6.

To confirm the association between UL76 and PAR, we conducted an immunoprecipitation assay using PAR antibody. Myc-tagged UL76 was transfected to HEK293T cells and the cell lysates were immunoprecipitated with PAR antibody or control IgG. Myc-UL76 could be immunoprecipitated by PAR antibody, which was affected when cells were treated with Olaparib to inhibit PAR synthesis (Figure 7A). This result was further verified by reciprocal immunoprecipitation assays. SFB-tagged UL76, UL24 and ORF20 were transfected to HEK293T cells and the cell lysates were immunoprecipitated with FLAG antibody. The results exhibited that only UL76 could pull down PAR, its analogs in HSV-1 (UL24) and KSHV (ORF20) did not bind to PAR chains (Figure 7B). To further map the minimum region responsible for UL76 binding to PAR, UL76 truncations were tested in a micro-irradiation assay for their ability to accumulate at the DNA damage strips. Neither the N terminal 1–150 aa fragment nor the 1–200 aa fragment of UL76 could accumulate at the DNA damage sites, and both 1–250 aa and 1–300 aa were clearly recruited to the DNA damage strips (Figure 7C). The kinetics of their accumulation were also recorded for 10 min post micro-irradiation (Figure 7D). The association between UL76 and PAR was also examined through an in vitro PAR overlay assay. About 20 μM of purified recombinant protein of GST or GST-tagged UL76 fragments were separated by SDS-PAGE and transferred to nitrocellulose membrane. The membrane was blocked and incubated with 100 nM purified PAR in TBS-T buffer for 1 h. After three stringent washing steps, the membrane was probed with the PAR antibody. Consistent with the micro-irradiation assay, no PAR signals were detected in the GST and GST-1–200 fragment of UL76 and PAR binding was detected in the GST-1–250 aa and 1–300 aa fragment of UL76 (Figure 7E). These observations demonstrated that UL76 directly bound to PAR chains and the PAR-binding motif of UL76 located in the 200–250 aa region.

After carefully analyzing the protein sequence from 200–250 aa of UL76, we found this region contains RG/RGG repeats (Figure 7F). RG/RGG repeats have been reported to mediate the binding of FUS or CIREB to PAR [53,54,55]. To determine whether these RG/RGG repeats were responsible for the binding between UL76 and PAR, the positively charged arginine residues in these RG/RGG repeats were mutated to alanine (8R/A) (Figure 7F). The recruitment of UL76 8R/A mutant on the DNA damage strips was dramatically reduced (Figure 7G,H), suggesting that UL76 bound to PAR chains through the RG/RGG repeats.

### 3.8. Inhibition or Depletion of PARP-1 Increases Type 1 IFN Response in HCMV Infected Cells

**Figure 8:**
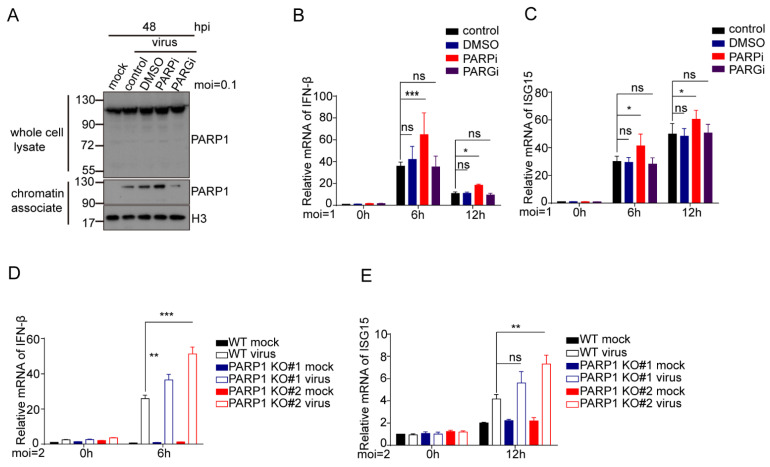
PARP-1 inhibition or depletion attenuates HCMV replication by promoting HCMV triggered induction of type Ⅰ IFNs. (**A**) PARP-1 inhibition increased HCMV infection-induced PARP-1 trapping on genomic DNA, while PARG inhibition decreased this trapping effect. MRC-5 cells were infected with HCMV at an MOI of 1. After adsorption, culture medium was replaced with fresh medium containing DMSO, 10 μM Olaparib, or 10 μM PARG inhibitor, respectively. Drugs were replenished every 24 h. At 48 hpi, the cells were collected and cell fractions were analyzed by Western blot with indicated antibodies. (**B**,**C**) PARP inhibition increased the transcription of *IFN-β* (**B**) and *ISG15* (**C**) in HCMV-infected MRC-5 cells at indicated times. MRC-5 cells were infected with HCMV at an MOI of 1 and treated as panel A. RNA was extracted and reverse transcribed to cDNA. Expression profiles of *IFN-β* and *ISG15* were analyzed by RT-qPCR. Data were analyzed by one-way ANOVA. * *p* < 0.05, ** *p* < 0.01, *** *p* < 0.001. (**D**,**E**) PARP-1 depletion upregulates the transcription of *IFN-β* (**D**) and *ISG15* (**E**) in HCMV-infected immortalized HFFs at indicated times. Wild type or PARP-1 depleted immortalized HFFs were infected with HCMV at an MOI of 2, and total RNA were extracted at indicated times. After being reverse transcribed to cDNA, the expression profiles of *IFN-β* and *ISG15* were analyzed by RT-qPCR. Data were analyzed by one-way ANOVA. * *p* < 0.05, ** *p* < 0.01, *** *p* < 0.001.

The immunomodulatory functions of PARP-1 inhibitors (PARPi) in cancers and inflammatory diseases have attracted increasing attention [56,57]. PARPi triggered innate immunity through the PARP-1 trapping-induced DNA damage response [58]. Recently, it has been reported that the pharmacological inhibition of PARP-1 significantly inhibits Pseudorabies virus (PRV, which belongs to the *Alphaherpesvirinae*) replication through DNA damage-induced innate antiviral immunity [59]. To understand the underlying mechanisms that HCMV replication was attenuated when PARP-1 activity was inhibited or PARP-1 was absent, we first evaluated the PARP-1 trapping effect induced by PARPi or PARGi in HCMV-infected cells. HCMV infection induced PARP-1 trapping on genomic DNA (Figure 8A). Moreover, the PARPi treatment increased PARP-1 trapping on genomic DNA, while PARGi treatment decreased this trapping effect. We also measured the type Ⅰ IFN responses in these cells. HCMV infection activates the transcription of *IFN-β* and *ISG15*. PARPi treatment in parallel with HCMV infection further increased the transcription of *IFN-β* and *ISG15* (Figure 8B,C). However, the PARGi treatment makes no difference to the transcription of these genes in HCMV-infected cells. During the preparation of this manuscript, Wang and colleagues demonstrated that PARP-1 negatively regulated the host’s innate antiviral immunity by PARylating cyclic GMP-AMP synthase (cGAS) and inhibited its DNA binding ability and subsequent immune responses upon HSV-1 infection [41]. We also compared the *IFN-β* and *ISG15* transcription in the WT and PARP-1-depleted HFF-TERTs infected with HCMV. PARP-1 depletion also elevated *IFN-β* and *ISG15* transcription upon HCMV infection (Figure 8D,E). The increased IFN genes transcription may contribute to the inhibitory effect of PARP-1 inhibition or depletion on HCMV infection.

## 4. Discussion

In the present study, we explored the role of PARP-1 in HCMV infection and found that HCMV infection activated PARP-1 and increased protein PARylation as early as 12 hpi (Figure 2A,B). PARP-1 is potently activated by DNA damage and facilitates DNA repair, and it has been well documented that both HSV-1 and HCMV infection activate certain DNA damage responses [7,8,60,61]. The DNA damage response (as measured by DNA damage hallmark, γH2AX) persisted along the HCMV infection course (Figure 1A and Figure S4D). It was suggested that replication/or resolution of the viral genome concatemers may result in nicks and breaks on the genome of HSV-1 [62]. These nicks and breaks could be recognized by PARP-1 and thus activate PARP-1. Virus infection also causes the generation of reactive oxygen species (ROS) and increased oxidative stress-induced DNA damage in host cells [63]. Thus, the activation of PARP-1 upon HCMV infection may result from ROS-induced host’s DNA damage or nicks and breaks on the incoming viral genome.

Most recently, a study from Wang and colleagues unveiled the role of PARP-1 in regulating the innate antiviral immune response against HSV-1 [41]. They showed that HSV-1 infection-induced DNA damage leads to phosphorylation and translocation of PARP-1. The repositioned PARP-1 PARylated cGAS and inhibited the DNA-binding activity of cGAS and subsequent antiviral response. We also found that PARP-1 was translocated from nucleus to cytoplasm upon HCMV infection (Figure 2C,D). Consequently, type Ⅰ IFN response was increased in the cells infected with HCMV when PARP-1 was inhibited or depleted (Figure 8). These results indicated that PARP-1 and its enzymatic activity may favor HCMV reproduction by inhibiting innate antiviral immunity. Moreover, our results showed that PARP-1 depletion caused more severe defects in HCMV infection than PARP-1 inhibition. This indicated that the important role of PARP-1 on HCMV infection might not be limited to PARP-1-mediated antiviral immunity. The PARP inhibitor, Olaparib, used in this study targets PARP-1 and PARP-2. Thus, whether PARP-2 is also involved in the HCMV infection process still needs further studies.

Recently, the requirement for PARP-1 activity to process unlinked Okazaki fragments has been reported [23]. It was widely accepted that viruses need the cooperation of host’s factors to facilitate their DNA replication. PARP-1 was enriched on HSV-1 and HCMV replication forks [64,65,66]. PARP-1 may also facilitate the viral replication by facilitating viral DNA replication processes. Consistent with this hypothesis, the colocalization of PARP-1 and HCMV DNA polymerase processivity factor UL44 (usually used to indicate the viral replication compartments) was evident in HCMV-infected cells (Figure 2C). We hypothesized that, during HCMV infection, viral DNA replication results in DNA nicks or gaps, which induces PARP-1 activation. Activated PARP-1 may facilitate the processing of unlinked Okazaki fragments during viral DNA replication. Collectively, PARP-1 may favor HCMV replication through both regulating the innate antiviral immune signaling and directly facilitating the viral DNA replication process.

In contrast to PARP-1, the role of PARG in viral replication was less reported. HSV-1 infection induced a proteasome-dependent degradation of the nuclear isoform of PARG which was mediated by the ICP0 of HSV-1 [33]. The knockdown of all of the PARG isoforms by siRNA resulted in a moderate decrease in HSV-1 yield, suggesting that PARG activity facilitates HSV-1 replication. In the context of HCMV infection, we found that HCMV infection upregulated the transcription of *PARG* in fibroblast, which was continuously increased from 12 hpi to 96 hpi (Figure 3A,B). The upregulation of PARG is independent on viral DNA replication (Figure 3D,E), indicating that some IE or E genes were responsible for the upregulation of PARG. The HCMV lytic replication cycle lasts for about 96 h [4]. During HCMV infection, PARP-1 may be continuously activated, and the activated PARP-1 produced a large amount of PAR polymers which could serve as a death signal to the host cells. The protracted replication cycle forces HCMV to maintain host cell viability for a relatively long period of time. The physiological importance of PARG is suggested by the rapid turnover of PAR in vivo. Upregulation of PARG may directly reduce the accumulation of PAR polymers, which protects host cells against PARP-dependent cell death. Adequate PARG made it possible for cells to timely degrade PAR polymers to free ADP-ribose.

Functionally, the inhibition of PARG after HCMV infection is beneficial to the production of HCMV, but does not affect the expression of type I interferon genes. Thus, we assumed that PARG inhibition may have extra functions besides regulating the innate antiviral immune response. Nonprotein PAR polymers degraded from acceptor proteins by PARG translocate from nucleus into the cytoplasm, where it mediates the releasing of apoptosis-inducing factor (AIF) and subsequent parthanatos [67]. The degradation of the nuclear isoform of PARG in mouse astrocytes has been shown to slow the rate of nuclear PAR degradation and protect against PARP-dependent cell death [68]. PARG inhibition may block the hydrolysis of PAR polymers from PARylated proteins and subsequent parthanatos, which in turn contribute to HCMV replication.

Although increased PARG levels prevented the accumulation of PAR chains, they could not prevent the sustained consumption of NAD^+^ caused by PARP-1 activation. We found that HCMV UL76 inhibited PARP-1 activity (Figure 5). UL76 is directly bound with the BRCT domain of PARP-1 (Figure 4F). As most auto-modification sites exist in the BRCT domain, UL76 may block the BRCT region to prevent PARP-1 auto-modification, which reduces the PARP-1 PARylation level. Furthermore, the association between PARP-1 and UL76 may directly affect the enzymatic activity of PARP-1 on other substrates, as the PARylation of histones was also affected by UL76 overexpression (Figure 5E). Given that HCMV infection resulted in PARP-1 activation, the “true late” protein UL76 may prevent the overactivation of PARP-1 during HCMV infection, which may avoid the dramatic change in NAD^+^ levels. As both decreases of NAD^+^ level and overgeneration of PAR chains led to cell death [69]. UL76 may functioned to balance the viral DNA replication and cell fate of infected cells. DNA damage and abnormal mitosis were reported in UL76 stably expressed cells [15,70], which could also result from PARP-1 inhibition [71]. Interestingly, UL24 and ORF20, two UL76 homologs from HSV-1 or KSHV, respectively, could not bind with PARP-1 and PAR chains, indicating that this unique regulation of protein PARylation was specific to HCMV infection. However, the exact role of UL76 in HCMV infection needs further evidence from UL76 deficient mutant. Protein PARylation levels in UL76 deficient mutant infected cells need to be analyzed. Whether UL76 affect the translocation of PARP1 induced by HCMV infection also need to be investigated. There are also other limitations to our study, such as only one laboratorial HCMV strain was tested. Extensive propagation in fibroblasts led to the deletion of several genes on AD169 genome. These genes not only affected viral cell tropism [72] but also influenced the innate immune response against HCMV [73]. The role of UL76 on PARP1 and PARylation should be examined in cell lines which are permissive for HCMV infection.

**Figure 9:**
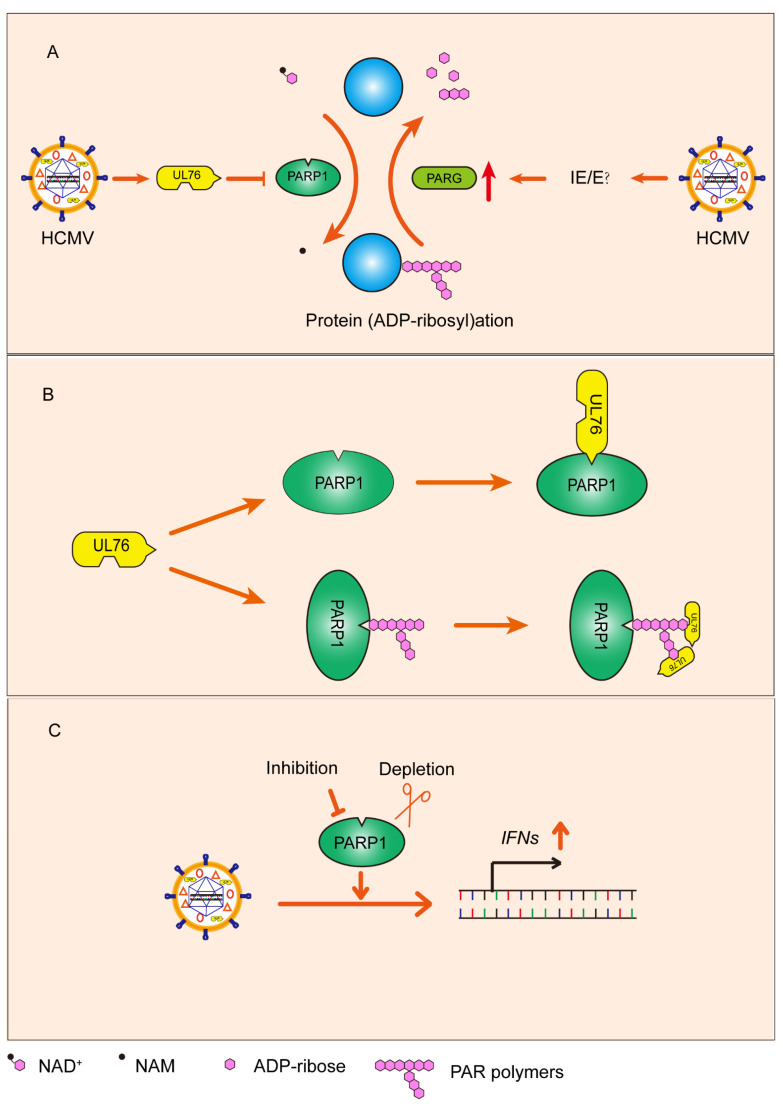
Simplified model summarizing the major findings from the present study. (**A**) HCMV UL76 combined with the infection induced PARG upregulation to manipulate protein PARylation in infected cells. (**B**) Under quiescent state, HCMV tegument protein UL76 bound to the BRCT domain of PARP-1 through its C terminal. When PARP-1 was activated, UL76 bound to PAR polymers via its RGG motifs. (**C**) Inhibition or depletion of PARP-1 in HCMV infected cells increased the type Ⅰ IFN response and decreased viral reproduction.

Collectively, we explored the role of PARP-1 and PARG in HCMV infection and uncovered the relationship between HCMV and protein PARylation (Figure 9A). HCMV infection activated PARP-1 and upregulated PARG. Some IE or E genes were responsible for the upregulation of PARG in HCMV infected cells. We also found that HCMV UL76 binds to the BRCT domain of PARP-1 and inhibits PARP-1 mediated protein PARylation (Figure 9B). HCMV UL76 and upregulated PARG coordinately downregulated protein PARylation which may prevent PARP-1 overactivation induced cell death. These observations implicated that HCMV infection actively regulated the protein PARylation to counteract challenges from the protracted replication cycle of HCMV. Promoting IFN gene expression contributes to the inhibitory effect of PARP-1 inhibition or depletion on HCMV replication (Figure 9C).

## Figures and Tables

**Table 1:** Primers used for plasmid construction.

Plasmid	Forward	Reverse
SFB-PARP1	acgcGTCGACggATGGCGGAGTCTTCGGATAAGC	ggaAGATCTTTACCACAGGGAGGTCTTAAAATTG
SFB-PARP2	ataGGCGCGCCTATGGCGGCGCGGCG	acgcGTCGACTCACCACAGCTGAAGGAAATTAAACTG
GFP-PARP1	acgcGTCGACggATGGCGGAGTCTTCGGATAAGC	ccgCTCGAGTTACCACAGGGAGGTCTTAAAATTG
pET30-PARP1	actGAGCTCcgATGGCGGAGTCTTCGGATAAGC	ccgCTCGAG CCACAGGGAGGTCTTAAAATTG
SFB-UL76	cgGAATTCATGCCGTCCGGGCGT	cgGGATCCCTATAAAGACCGTGTGGGAC
myc-UL76	cgGAATTCggATGCCGTCCGGGCGT	cgGGTACCCTATAAAGACCGTGTGGGAC
GFP-UL76	cccAAGCTTcgATGCCGTCCGGGCGT	cgGGATCCCTATAAAGACCGTGTGG
SFB-UL24	ttGGCGCGCCTATGGCCGCGAGAACG	acgcGTCGACTCATTCGGAGGCGGCT
SFB-ORF20	ttGGCGCGCCTATGGTACGTCCAACCG	acgcGTCGACTCATGGACCTGAACAAGC
GFP-IE1	cgGAATTCtATGGAGTCCTCTGCCA	cgGGATCCTTACTGGTCAGCCTTGCTTC
GFP-pp65	ccgCTCGAGctATGGAGTCGCGCGGTC	cgGGATCCTCAACCTCGGTGCTTTTTGG
GFP-UL24	cccAAGCTTcgATGGCCGCGAGAACG	cgGGATCCTCATTCGGAGGCGGCT
GFP-ORF20	cccAAGCTTcgATGGTACGTCCAACCG	cgGGATCCTCATGGACCTGAACAAGC
pGEX4T-UL76	cgGGATCCATGCCGTCCGGGCGT	cgCTCGAGCTATAAAGACCGTGTGGGAC
PARP1 E988K	ATCTGTTACATGAACAATATATCATCTCTCC	TAGACAATGTACTTGTTATATAGTAGAGAGG
SFB-PARP1 DN	acgcGTCGACggGTGGATGAAGTGGCGAA	ggaAGATCTTTACCACAGGGAGGTCTTAAAATTG
SFB-PARP1 DM	F1:acgcGTCGACggATGGCGGAGTCTTCGGATAAGC R1:GGCCACAACTTCAACAGGTCCATCCACCTCATCGC	F2:GGCGATGAGGTGGATGGACCTGTTGAAGTTGTGG R2:ggaAGATCTTTACCACAGGGAGGTCTTAAAATTG
SFB-PARP1 DW	F1:acgcGTCGACggATGGCGGAGTCTTCGGATAAGC R1:CACTGCCTCTTCATCCTGGTTGATACCTTCCTCCTTG	F2:TCAAGGAGGAAGGTATCAACCAGGATGAAGAGGCAG R2:ggaAGATCTTTACCACAGGGAGGTCTTAAAATTG
SFB-PARP1 DC	acgcGTCGACggATGGCGGAGTCTTCGGATAAGC	ggaAGATCTTCAGCCAGGATTTACTGTCAGC
SFB-PARP1 DZ	F1:acgcGTCGACggATGGCGGAGTCTTCGGATAAGC R1:CACAGCAGCAGGAGCCGATCCATCCACCTCATCGC	F2:CGATGAGGTGGATGGATCGGCTCCTGCTGCT R2:ggaAGATCTTTACCACAGGGAGGTCTTAAAATTG
SFB-PARP1 DB	F1:acgcGTCGACggATGGCGGAGTCTTCGGATAAGC R1:GGCCACAACTTCAACAGGGGCTGTGGAGGGCGGA	F2:CCTCCGCCCTCCACAGCCCCTGTTGAAGTTGTGGCCC R2:ggaAGATCTTTACCACAGGGAGGTCTTAAAATTG
GFP-UL76 1-150	cccAAGCTTcgATGCCGTCCGGGCGT	cgGGATCCCTAGACTTGACCGCCACCG
GFP-UL76 1-200	cccAAGCTTcgATGCCGTCCGGGCGT	cgGGATCCCTAAAGGTGTGCAACAGACTCATC
GFP-UL76 1-250	cccAAGCTTcgATGCCGTCCGGGCGT	cgGGATCCCTACGCACCCGGTCCATGA
GFP-UL76 1-300	cccAAGCTTcgATGCCGTCCGGGCGT	cgGGATCCCTATGTAGCAGCGTCCGCG

## Data Availability

All data generated or analyzed during this study are included in this published article.

## Supplementary Materials

The following supporting information can be downloaded at: https://www.mdpi.com/article/10.3390/v14092049/s1, Figure S1. PARP-1 inhibition and PARG inhibition showed different effects on HCMV replication; Figure S2. Specificity of the UL76 antibody in detecting ectopic expressed or viral encoded UL76; Figure S3. Analysis of the binding between UL76 and PARP-1; Figure S4. Generation of RPE cells with inducible SFB-tagged UL76 overexpression.

## Abbreviations

PARP-1: Poly(ADP-ribose) polymerase 1; PARG: Poly(ADP-ribose) glycohydrolase; PARylation: Poly(ADP-ribosyl)ation; HCMV: Human Cytomegalovirus; HSV-1: Herpes simplex virus 1; KSHV: Kaposi’s sarcoma-associated herpesvirus; BRCT: BRCA C terminal; γH2AX: phosphorylated histone 2A variant X; cGAS: cyclic GMP-AMP synthase; hTERT: human telomerase reverse transcriptase; IFN: Interferon; PFA: Phosphonoformic acid; ISG: Interferon-stimulated gene; AIF: apoptosis inducing factor.
