# Supplementary material for: Role of PARP-1 in Human Cytomegalovirus Infection and Functional Partners Encoded by This Virus
Source: Viruses. 2022 Sep 15;14(9):2049. doi: 10.3390/v14092049 (PMC9501325; doi:10.3390/v14092049)
Supplement: Supplementary file 1 [file viruses-14-02049-s001.zip › viruses-1881059-supplementary.pdf]

Supplementary figures

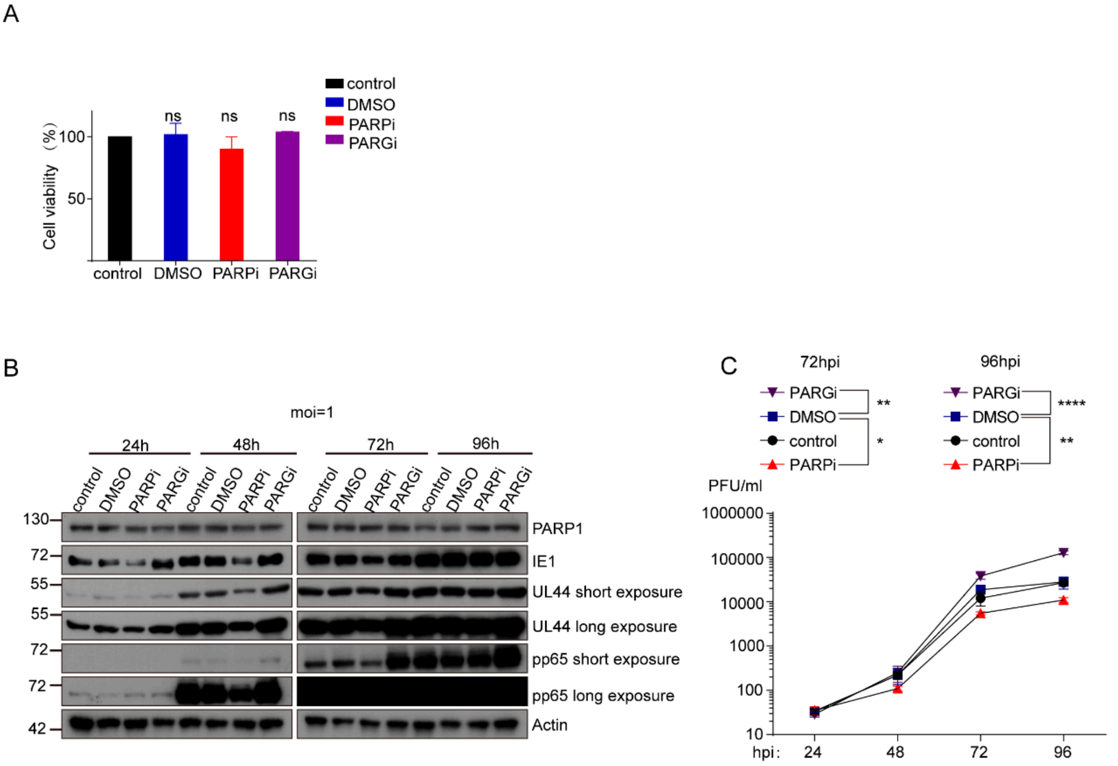

Figure S1. PARP-1 inhibition and PARG inhibition showed different effects on HCMV replication. **(A)** MRC-5 cells were treated with PARPi (10 μM), PARGi (10 μM) or an equal volume of DMSO for 48h and the cell viability was assessed with CCK-8 assays. **(B)** PARP1 activity was required for efficient HCMV replication. MRC-5 cells were infected with HCMV at an MOI of 1. After adsorption, the cells were refed with fresh medium containing DMSO, 10μM Olaparib or 10μM PARG inhibitor. The effects of PARPi and PARGi on HCMV replication were analyzed by WB. **(C)** Single-step growth curves of HCMV in PARPi or PARGi treated MRC-5 cells. MRC-5 were infected with HCMV AD169 strain at an MOI of 1 and treated as panel B. The culture supernatants were collected at the indicated times post-infection, and infectious virus titers were determined by the standard plaque formation assay. The mean values are shown with bars denoting standard error for two independent experiments. *, $p<0.05$; **, $p<0.01$; ****, $p<0.0001$

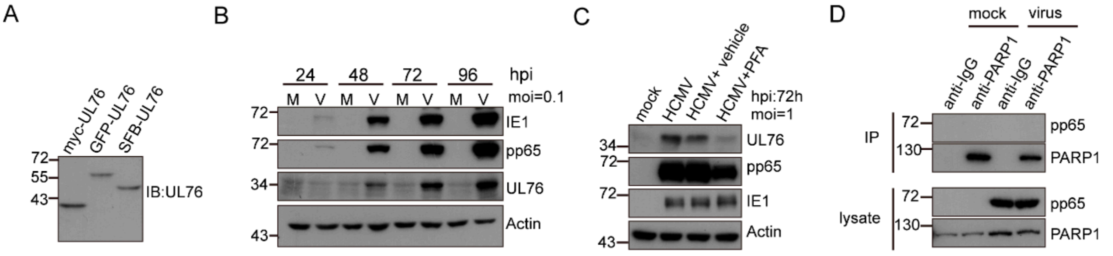

Figure S2. Specificity of the UL76 antibody in detecting ectopic expressed or viral encoded UL76. **(A)** A rabbit polyclonal antibody to HCMV UL76 was raised against the full length of UL76. Myc-UL76, GFP- UL76, SFB-UL76 were transfected to HEK293T cells. The cells lysates

were separated and transferred to PVDF membrane. The membrane was blocked and probed with this UL76 antibody. **(B)** The expression profile of UL76 in HCMV reproductive replication cycle. MRC-5 cells were infected with HCMV at an MOI of 0.1. The expression of UL76, IE1 and pp65 at indicated time points post-infection were analyzed by western blot. **(C)** The expression of UL76 was significantly decreased when viral DNA replication was inhibited by PFA. The MRC-5 cells were infected with HCMV at an MOI of 1. After adsorption, 250 ug/ml of PFA or vehicle were added to the medium. Cells were harvested at 72 hpi and expression of UL76, pp65 and IE1 were analyzed by WB. **(D)** No binding between PARP1 and HCMV pp65 was detected in HCMV infected cells by coimmunoprecipitation assay. The MRC-5 cells were infected or mock infected with HCMV at an MOI of 1 for 48h. Cell lysates were prepared and subjected to immunoprecipitation with PARP-1 antibody or control IgG.

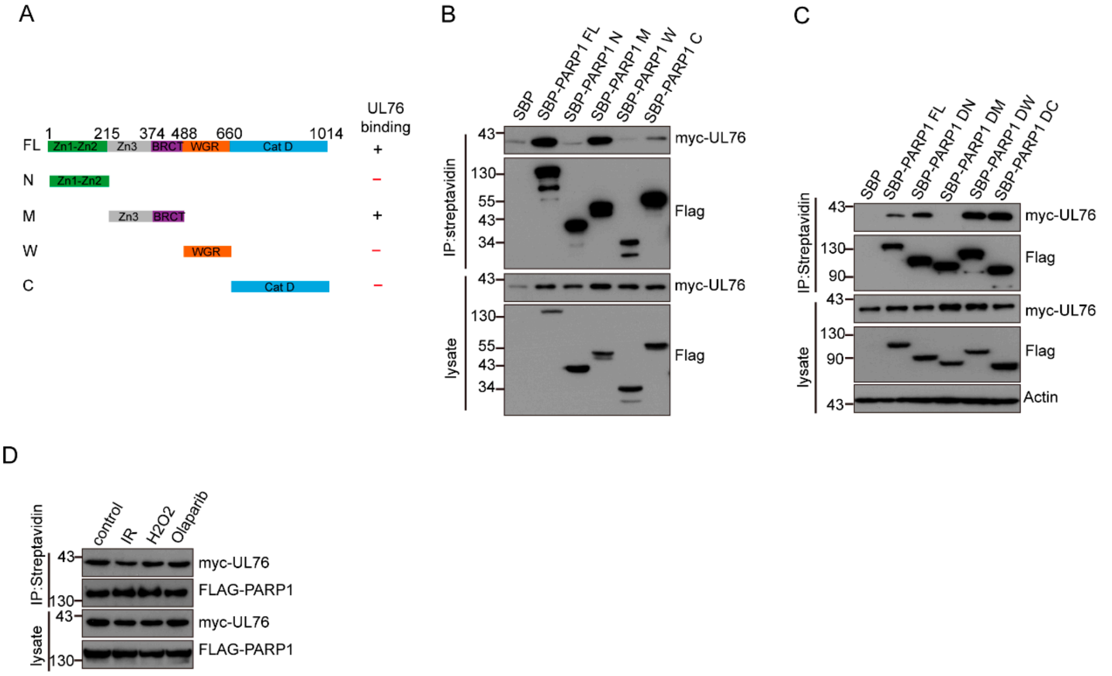

Figure S3. Analysis of the binding between UL76 and PARP-1. **(A)** Schematic depiction of PARP-1 and its truncate mutants. Results from domain mapping experiments indicated that UL76 binds to the auto-modification domain of PARP-1. **(B)** HEK293T cells were co-transfected with myc-tagged UL76 and SFB-tagged PARP-1 truncations. The cells were collected 48h post-transfection and subjected to streptavidin precipitation. **(C)** The auto-modification domain deletion mutant of PARP-1 failed to interact with UL76. HEK293T cells were co-transfected with myc-tagged UL76 and SFB-tagged PARP-1 WT, or PARP-1 deletion mutants. Cell lysates were subjected to precipitation and analyzed by immunoblotting with indicated antibodies. **(D)** Activation or inhibition of PARP-1 activity does not affect the association between UL76 and PARP-1. SFB-PARP-1 and myc-UL76 were co-transfected to HEK293T cells. Cells were treated with 1 mM H_2O_2 for 5 min or exposed to irradiation (5Gy) to activate PARP-1. Cells were treated with Olaparib (10 μM) for 30 min to inhibit PARP-1 activity.

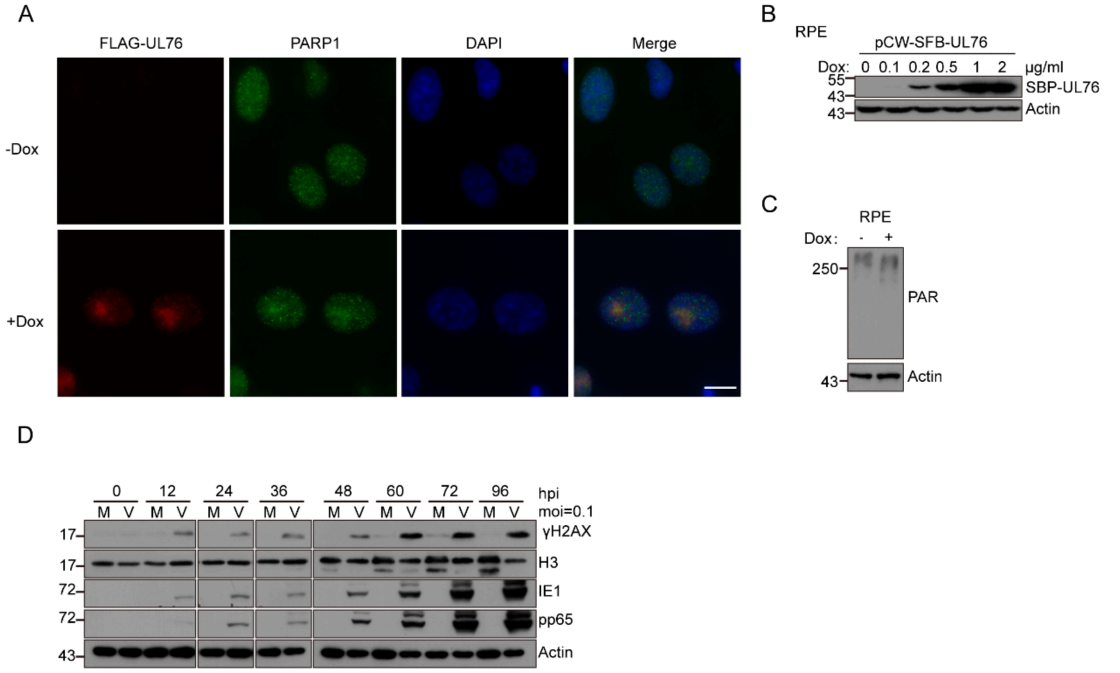

Figure S4. Generation of RPE cells with inducible SFB-tagged UL76 overexpression. **(A)** Inducible expressed SFB-UL76 colocalized with endogenous PARP-1 in RPE cells lines. SFB-UL76 were cloned to downstream of a Tet-On promotor and introduced to RPE cells. Puromycin resistant clones were selected and tested for the inducible expression of UL76. Cells were induced with or without 1 μg/ml doxycycline for 24h. The expression and distribution of SFB-UL76 and PARP1 were analyzed by immuneflouresence assay. The cell nuclei were counterstained with DAPI. Scale bar, 10 μm. **(B)** The Tet-On inducible UL76 RPE cells were induced with increasing amount of Dox for 24h and the expression of UL76 were tested by WB. A single band with a predicted MW of 50KD was detected. The expression of UL76 correlated positively with the increasing concentration of Dox. **(C)** Dox induction has no effect on protein PARylation levels in WT RPE cells. To exclude the possibility that Dox treatment affected protein PARylation. Wild type RPE cells were induced with or without Dox for 24h and protein PARylation were analyzed by WB. **(D)** HCMV infection triggered DNA damage response. MRC-5 cells were infected with HCMV at an MOI of 0.1. Cells were harvested at indicated times and phosphorylation levels of histone H2AX (γH2AX) were analyzed by WB.
